# Management Measures and Trends of Biological Invasions in Europe: A Survey‐Based Assessment of Local Managers

**DOI:** 10.1111/gcb.70028

**Published:** 2025-01-17

**Authors:** Carla Garcia‐Lozano, Josep Pueyo‐Ros, Quim Canelles, Guillaume Latombe, Tim Adriaens, Sven Bacher, Ana Cristina Cardoso, Michelle Cleary, Lluís Coromina, Franck Courchamp, Wayne Dawson, Maarten de Groot, Franz Essl, Belinda Gallardo, Marina Golivets, Erja Huusela, Miia Jauni, Sven D. Jelaska, Jonathan M. Jeschke, Stelios Katsanevakis, Melina Kourantidou, Ingolf Kühn, Bernd Lenzner, Brian Leung, Elizabete Marchante, Colette O'Flynn, Cristian Pérez‐Granados, Jan Pergl, Pavel Pipek, Cristina Preda, Filipe Ribeiro, Helen Roy, Riccardo Scalera, Menja von Schmalensee, Hanno Seebens, Róbert A. Stefánsson, Barbara Tokarska‐Guzik, Elena Tricarico, Sonia Vanderhoeven, Vigdis Vandvik, Montserrat Vilà, Núria Roura‐Pascual

**Affiliations:** ^1^ Departament de Geografia Universitat de Girona Girona Spain; ^2^ Departament de Ciències Ambientals, Facultat de Ciències Universitat de Girona Girona Spain; ^3^ Catalan Institute for Water Research (ICRA‐CERCA) Girona Spain; ^4^ Institute of Ecology and Evolution, the University of Edinburgh, King's Buildings Edinburgh UK; ^5^ Research Institute for Nature and Forest (INBO) Brussels Belgium; ^6^ Department of Biology University of Fribourg Fribourg Switzerland; ^7^ European Commission Joint Research Centre (JRC) Ispra Italy; ^8^ Swedish University of Agricultural Sciences (SLU) Southern Swedish Forest Research Centre Alnarp Sweden; ^9^ Facultat de Turisme Universitat de Girona Girona Spain; ^10^ Centre National de Recherche Scientifique, AgroParisTech, Ecologie Systématique Evolution, Université Paris‐Saclay Gif‐Sur‐Yvette France; ^11^ Department of Evolution, Ecology and Behaviour Institute of Infection, Veterinary, and Ecological Sciences, University of Liverpool Liverpool UK; ^12^ Slovenian Forestry Institute Ljubljana Slovenia; ^13^ Division of BioInvasions, Global Change & Macroecology, Department of Botany and Biodiversity Research University of Vienna Vienna Austria; ^14^ Instituto Pirenaico de Ecología (IPE), CSIC Zaragoza Spain; ^15^ Department of Community Ecology Helmholtz Centre for Environmental Research ‐ UFZ Halle Germany; ^16^ Natural Resources Institute Finland (Luke) Jokioinen Finland; ^17^ Natural Resources Institute Finland (Luke) Helsinki Finland; ^18^ Faculty of Science University of Zagreb Zagreb Croatia; ^19^ Leibniz Institute of Freshwater Ecology and Inland Fisheries (IGB) Berlin Germany; ^20^ Institute of Biology Freie Universität Berlin Berlin Germany; ^21^ Berlin‐Brandenburg Institute of Advanced Biodiversity Research (BBIB) Berlin Germany; ^22^ Department of Marine Sciences University of the Aegean Mytilene Greece; ^23^ Université de Bretagne Occidentale, AMURE, IUEM ‐ Institut Universitaire Européen de la Mer Plouzané France; ^24^ Department of Business and Sustainability University of Southern Denmark Business School Esbjerg Denmark; ^25^ Geobotany and Botanical Garden, Martin Luther University Halle‐Wittenberg Halle Germany; ^26^ German Centre for Integrative Biodiversity Research (iDiv) Halle‐Jena‐Leipzig Leipzig Germany; ^27^ Department of Biology McGill University Montreal Quebec Canada; ^28^ Bieler School of Environment McGill University Montreal Quebec Canada; ^29^ Department of Life Sciences, Centre for Functional Ecology, Associate Laboratory TERRA University of Coimbra Coimbra Portugal; ^30^ National Biodiversity Data Centre Waterford Ireland; ^31^ Ecology Department Alicante University Alicante Spain; ^32^ Biodiversity Management and Conservation Programme, Forest Science and Technology Centre of Catalonia Solsona Spain; ^33^ Institute of Botany, Czech Academy of Sciences Průhonice Czech Republic; ^34^ Department of Ecology, Faculty of Science Charles University Prague Czech Republic; ^35^ Ovidius University of Constanta Constanta Romania; ^36^ MARE, Centro de Ciências do Mar e do Ambiente Faculdade de Ciências, Universidade de Lisboa Lisbon Portugal; ^37^ UK Centre for Ecology & Hydrology Wallingford UK; ^38^ Centre for Ecology and Conservation University of Exeter Penryn UK; ^39^ IUCN/SSC Invasive Species Specialist Group Rome Italy; ^40^ West Iceland Nature Research Centre Stykkishólmur Iceland; ^41^ Department of Animal Ecology & Systematics Justus‐Liebig University Giessen Giessen Germany; ^42^ Institute of Biology, Biotechnology and Environmental Protection, University of Silesia in Katowice Katowice Poland; ^43^ Department of Biology University of Florence Sesto Fiorentino Italy; ^44^ Belgian Biodiversity Platform Service Public de Wallonie Namur Belgium; ^45^ Department of Biological Sciences University of Bergen Bergen Norway; ^46^ Estación Biológica de Doñana (EBD‐CSIC) Sevilla Spain; ^47^ Department of Plant Biology and Ecology University of Sevilla Sevilla Spain

**Keywords:** environmental perception, exotic species, expert survey, invasive alien species, management practices, non‐native species, policy

## Abstract

Biological invasions are a major threat to biodiversity, ecosystem functioning and nature's contributions to people worldwide. However, the effectiveness of invasive alien species (IAS) management measures and the progress toward achieving biodiversity targets remain uncertain due to limited and nonuniform data availability. Management success is usually assessed at a local level and documented in technical reports, often written in languages other than English, which makes such data notoriously difficult to collect at large geographic scales. Here we present the first European assessment of how managers perceive trends in IAS and the effectiveness of management measures to mitigate biological invasions. We developed a structured questionnaire translated into 18 languages and disseminated it to local and regional managers of IAS in Europe. We received responses from 1928 participants from 41 European countries, including 24 European Union (EU) Member States. Our results reveal substantial efforts in IAS monitoring and control, with invasive plants being the primary focus. Yet, there is a general perception of an increase in the numbers, occupied areas, and impacts of IAS across environment and taxonomic groups, particularly plants, over time. This perceived increase is consistent across both EU and non‐EU countries, with respondents from EU countries demonstrating more certainty in their responses. Our results also indicate a lack of data on alien vertebrates and invertebrates, reflecting a need for more targeted monitoring and knowledge sharing between managers and policymakers and between countries. Overall, our study suggests that Europe's current strategies are insufficient to substantially reduce IAS by 2030 and hence to meet the Kunming‐Montreal Global Biodiversity Framework target.

## Graphical abstract

Our study explores how managers across Europe perceive the trends and management of invasive alien species. Through a survey of nearly 2000 managers from 41 countries, we found substantial efforts dedicated to monitoring and controlling IAS, particularly invasive plants. However, managers expressed limited knowledge about invasive vertebrates and invertebrates. Despite ongoing efforts, most respondents believe that numbers, area, and impacts of IAS are increasing. This highlights that Europe's current strategies are insufficient to effectively curb the growing threat of biological invasions.

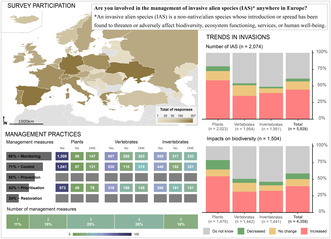

## 1 Introduction

Invasive alien species (hereafter abbreviated as IAS) threaten biodiversity, disrupt native communities, and impact the functioning of ecosystems (IPBES 2023). These changes negatively affect the benefits and services that ecosystems provide to humanity, often resulting in substantial costs related to the damages caused by biological invasions and their management (Ahmed et al. 2023; Bacher et al. 2023). Funding for the management of IAS has increased over the last decade (Cuthbert et al. 2022). However, the number of IAS being introduced to new regions continues to increase worldwide for most taxonomic groups and shows no signs of slowing down in the future (Seebens et al. 2021). With considerable variation in the ability of countries to address this global threat (Early et al. 2016; Latombe et al. 2023), further expansion of IAS, with ongoing environmental and climate change, is expected (Gallardo et al. 2017; Liu et al. 2023; Walther et al. 2009).

In response to the threat posed by biological invasions, several global initiatives have emerged over the last 30 years to document and manage IAS (see fig. 1.2 in IPBES (2023)). The recent Kunming‐Montreal Global Biodiversity Framework of the Convention of Biological Diversity (CBD) has set an ambitious target (Target 6) to eliminate, reduce, and mitigate impacts through pathway management, prevention and with a focus on priority species and priority sites (CBD 2022). Aligned with this global target, the European Union (EU) Biodiversity Strategy for 2030 aims to step up the implementation of the EU Regulation 1143/2014 on the prevention and management of the introduction and spread of IAS (European Union 2014) and other relevant legislation and international agreements. The objective is to manage IAS and decrease the number of Red List species they threaten in the EU (European Commission 2021a). While these initiatives are commendable in scope and ambition, the paucity of information about the management measures being implemented, their effectiveness in terms of reducing the rates of new species introductions and IAS impacts hinders the ability to assess progress and develop effective strategies to achieve these targets (Hulme 2024; Roura‐Pascual et al. 2024).

At the European level, several national and pan‐European efforts have contributed to improving the exchange of IAS management information (Katsanevakis et al. 2015, 2014; Lucy et al. 2016; Oficialdegui et al. 2023; Piria et al. 2017; Roy, Rabitsch, and Scalera 2018; Roy et al. 2019; Trichkova et al. 2017). The adoption of the EU Regulation 1143/2014 has also required EU Member States to provide detailed information on the distribution, spread, and reproductive patterns of IAS of Union concern, as well as the effectiveness of relevant management measures, especially on control and rapid eradication (see Article 24, European Union 2014). This information is made available in the European Alien Species Information Network (EASIN), a dedicated system supporting the implementation of the EU IAS Regulation (Tsiamis et al. 2016). Comprehensive online resources notifying early eradications (Notsys; https://easin.jrc.ec.europa.eu/notsys) and detailing management (measures and cost‐effectiveness) are currently available for nearly all IAS of Union concern and other IAS (https://iucn.org/our‐work/topic/invasive‐alien‐species/invasive‐alien‐species‐additional‐external‐resources), as well as on the management of alien vertebrates with animal welfare considerations (Smith et al. 2022).

Although this has made management information more comprehensive than ever before (e.g., European Commission 2021b), the data and reports provided by EU Member States are limited to specific, well‐monitored species or areas of interest (Cardoso et al. 2021; Polce et al. 2023) and remain inconsistent both in format and the qualitative and quantitative information they provide (available at EIONET Central Data Repository, under folders European Union (EU) obligations—Invasive alien species (1143/2014/EU); European Environmental Agency 2023). Additionally, not all EU Member States have contributed to these reports and information from other non‐EU states are not included, further limiting the scope and completeness of the available information on IAS at the European level. A major challenge arises from the fragmented and hierarchical nature of IAS management reporting, reliant on the acquisition of data by local and regional managers and the uptake of this information by the administrative structure for harmonization and integration into publicly available information systems. Without a centralized reporting system, it becomes difficult to track trends and evaluate management efficiency at continental scales (Gatto et al. 2013; Reyserhove et al. 2022).

In the absence of publicly available data on management practices across Europe, this study assesses the implementation and indirectly the effectiveness of IAS management at the European level, from the perspective of local and regional managers dealing with IAS. Managers' knowledge, while influenced by individuals' experience and environment (Shackleton et al. 2019b), provides a valuable understanding of how management is progressing. Asking managers' opinions about their practices allows for the inclusion of qualitative information and personal observations, which would otherwise be lost. Perceptions of those involved in IAS management can be an indicator of management effectiveness at local scales and might fill important knowledge gaps in management implementation (Shackleton et al. 2019a).

To assess managers' perception of IAS management practices and trends in Europe, we created and distributed a structured survey to managers across both EU Member States and non‐member countries, enabling a comparison of countries affected by the EU Regulation on IAS with those outside its influence. We asked IAS managers to: (1) estimate the efforts of management measures in their area of interest; (2) assess the trends of IAS in terms of the number of species, the area occupied, and their impacts, identifying differences between taxonomic groups and environments; and (3) evaluate the effectiveness of management measures on IAS trends. The survey was designed to cover all terrestrial and marine habitats, all organismal types, and a wide range of management options. It represents the first comprehensive evaluation of the perceived IAS trends and effectiveness of IAS management practices at a continental scale. For previous similar studies that are more limited in scope, see Andreu, Vilà, and Hulme (2009) and Paganelli et al. (2021). The information provided in this study offers insights into the approaches currently developed by European countries and specifically in the context of progress toward the Kunming‐Montreal Global Biodiversity Framework Target 6 of substantially reducing the impacts of IAS.

## 2 Methods

### 2.1 Survey Design

The survey was designed following (Dillman 2007; Dillman, Smyth, and Christian 2014; Krosnick 2018). It was developed by a group of researchers (*n* = 21) (Appendix S1: Table S1.1) and tested with a selected group of IAS managers (*n* = 5), whose feedback on both content and user experience was incorporated in the final version. The survey comprised a total of 27 closed‐ended questions, divided into 11 context‐related questions and 16 management‐related questions (Appendix S1: Table S1.2). The context‐related questions should verify the respondents' eligibility (Q1, Appendix S2) and gather information about their location (country, region; Q2, Q3), the managed environment (Q4), the study area (funding, size, level of protection; Q20, Q22–Q24) and the respondent's profile (affiliation, educational level, experience; Q25–Q27). For the focal environment (Q4), respondents had to choose among ten environments (multiple answers possible): urban areas, forests, grasslands, croplands, rivers, lakes and inner wetlands, hydraulic structure, terrestrial coastal areas, marine coastal areas, and oceans.

The management‐related questions aimed to characterize management practices and perceptions of IAS trends in their area of interest from 2015 to 2022. The date of the entry into force of the EU IAS Regulation was taken as the reference for both EU and non‐EU Member States. Management measures were formulated in the broadest sense, indicating whether certain actions concerning invasions (i.e., monitoring, prioritization, prevention, eradication, control, or restoration; Box 1) were undertaken or not (“Yes,” “No,” “Do not know”; Q9–Q19, Q21). Trends indicated changes in IAS numbers (i.e., species richness), the area occupied by IAS, and their impacts on biodiversity in the area of interest as perceived by the respondents (“Decrease,” “No change,” “Increase,” “Do not know”; Q5, Q6, Q8). Additionally, we enquired about the type of impacts (on biodiversity, economic activities, human health, and ecosystem services) caused by IAS (“Yes,” “No,” “Do not know”; Q7).

BOX 1 **Definitions of management measures and environments used in the manuscript.**

*Management measures* (These definitions are, wherever possible, aligned with the terminology used by the CBD and EU IAS Regulation. Although monitoring cannot be considered a form of active measure (Robertson et al. 2020), it has been included in the analysis because it supports direct management interventions and is an integral part of any management strategy.)

- Monitoring. Field work to detect and report new IAS, surveillance of existing invaded sites, detection of new invaded sites, surveillance of pathways, etc.
- Prioritization. Evaluation of IAS risks, establishment of management priorities regarding the species and sites where measures should be taken, etc.
- Prevention. Measures to prevent the introduction and spread of IAS such as public awareness activities, bans of/taxes for IAS possession, etc.
- Control. Measures to control established populations by means of physical, chemical or biological actions, etc.
- Eradication. Measures to eradicate newly introduced IAS or established populations, differentiating between complete and partial eradication depending on their success: Complete eradication refers to the removal of all populations of an IAS from the whole area being managed, while partial eradication refers to the removal of only a few populations. Rapid eradication refers to measures aiming to remove newly introduced IAS before they establish in the area.
- Restoration. Measures to improve the environmental quality of sites after IAS removal, for example, planting of native species or decontamination of water bodies, soils rehabilitation, etc.

*Environments*

- Marine environments. Category of environments that include coastal areas (intertidal zones, estuaries, and shelves) and oceans and seas.
- Continental environments. Category of environments that includes all land‐based environments, both terrestrial and freshwater. This category has been further subdivided into the following subcategories:
  ○ Urban areas are a special case of terrestrial environments, but they are distinguished by their particularity in driving invasions.
  ○ Terrestrial environments include wooded habitats (forests, shrublands, and sparsely vegetated land), grasslands, and croplands.
  ○ Freshwater environments comprise rivers, lakes, inner wetlands, and hydraulic structures (channels and dams).
  ○ Coastland environments consist of terrestrial coastal areas (cliffs, salt marshes, lagoons, dry beaches, and dunes).

Most management‐related questions could be answered separately for taxonomic groups (Q5, Q6, Q8, Q11, Q12, Q14, Q15, Q17–Q19) and for two broad environmental categories, based on respondents' selections in Q4 (Q5–Q8, Q11–Q15, Q17–Q19). The taxonomic groups considered were plants, invertebrates, vertebrates and others. The categories of environments were: continental (including urban areas, forest, grasslands, croplands, rivers, lakes and inner wetlands, hydraulic structure, or terrestrial coastal areas) and marine (comprising marine coastal areas and oceans exclusively) (Appendix S1: Panel S1.1). We chose to focus on the marine environment because there is considerably less information available on biological invasions in marine environments compared to terrestrial and freshwater invasions (Watkins et al. 2021), and because IAS management is more costly in the marine realm (due to this realm's high environmental connectivity) and requires different approaches to those used in terrestrial and freshwater environments (Macêdo et al. 2024).

The first four questions (Q1–Q4) were mandatory for respondents to answer in order to verify their eligibility and provide information about their location and environment. All other questions were optional, and respondents could skip them if they were not applicable or did not want to respond. To ensure consistency and avoid including participants who answered only the initial questions, we examined the response rate across the survey and retained only those participants who completed nearly all the questions (up to Q25). Responses “Not applicable” and unanswered questions were excluded from the analyses. Responses “Do not know” were used as a surrogate of uncertainty: a high frequency of such responses reflects a greater degree of uncertainty. Accordingly, in the sections presenting and discussing the results of the survey, the term “uncertainty” specifically refers to this aspect.

### 2.2 Dissemination of the Survey

The survey was translated into 18 languages and incorporated into the survey application (1KA 2021) for distribution. It was disseminated online across Europe via (i) the distribution lists of networks and organizations specialized in biological invasions (Appendix S1: Table S1.3), and (ii) collaborators functioning as national and/or regional distribution nodes in 24 European countries, of which 18 were EU Member States (Appendix S1: Table S1.1). The collaborators identified potential respondents in their respective countries and contacted them via email, requesting their participation in the survey. To monitor response rates, we linked responses to an online map that allowed collaborators to track variations in real time across their respective countries (Appendix S1: Figure S1.1). As the survey progressed, collaborators modified their efforts to dedicate more resources toward regions with lower response rates to minimize geographic bias. The survey was conducted over 11 months, from February to December 2022.

Our target respondents included IAS practitioners from various sectors, including public administration (natural protected areas and local, supramunicipal, regional, and national administration), nongovernmental organizations (NGOs), or nonprofit organizations (NPOs), the private commercial sector, and research institutions. The survey was aimed at executive managers (directors), mid‐level managers (team leaders), and technicians. The objective was to ensure that our survey reached a diverse and representative sample of expertise involved in regional and local IAS management across Europe. Due to the lack of a distinct natural divide between Europe and Asia, this study defined Europe's eastern boundary as extending from the Ural Mountains to the Caspian Sea. This resulted in the inclusion of countries such as Russia and Kazakhstan, as well as Turkey and Azerbaijan. This document, as well as any data and map included herein, are without prejudice to the status of or sovereignty over any territory, to the delimitation of international frontiers and boundaries and to the name of any territory, city, or area.

### 2.3 Data Preparation and Analysis

Due to the dependency of some responses on the environment and the taxonomic group, the number of responses and consequently the datasets used in subsequent analyses varied. Three different datasets were employed: (i) *the original dataset*, comprising as many entries as respondents in the survey, was employed for questions independent of the environment and the taxonomic group; (ii) *the continental* vs. *marine dataset*, including responses to continental, marine, and both continental and marine environments separated into independent responses, allowed us to explore differences between taxonomic groups for continental and marine environments; and (iii) *the specific dataset*, containing responses only from participants who selected a single environment (or similar type of environments) in Q4, which enabled us to examine differences between taxonomic groups for distinct, more specific environments. In particular, we distinguished between urban areas, terrestrial (considering wooded habitats, grasslands, and croplands), freshwater (comprising rivers, lakes and inner wetlands, and hydraulic structures), coastland (referring to terrestrial coastal areas), and marine environments (including marine coastal areas and oceans) (Appendix S1: Panel S1.1).

We summarized participants' responses regarding management measures and perceived IAS trends separately using heatmaps and bar plots. Bar plots illustrated the proportion of respondents involved in each management measure (monitoring, control, prevention, prioritization, and restoration) using the original dataset, while heatmaps displayed the number of respondents conducting monitoring, control and prioritization separated by taxonomic groups (plants, vertebrates, and invertebrates) using the continental vs. marine dataset. Additionally, we used bar plots to assess trends in the number of IAS, the area occupied by IAS, and the impacts of IAS on biodiversity across taxonomic groups using the continental vs. marine dataset. We also plotted the proportion of impact from IAS on different sectors using the original dataset. Finally, heatmaps depicted the relationship between trends in the number of IAS and their impact on biodiversity (categorized as increase, no change, decrease, and do not know) across taxonomic groups and categories of the environment (marine, freshwater, terrestrial, and urban), using the specific dataset.

To assess the relationships between management measures and IAS trends across environments and taxonomic groups, we performed Multiple Correspondence Analyses (MCA) for categorical variables (Greenacre 2007; Lebart, Morineau, and Warwick 1984) using the R package *FactoMineR* version 2.9 (Lê, Josse, and Husson 2008). These analyses, based on the continental vs. marine dataset, explored if perceived trends in IAS numbers, area occupied by IAS, and IAS impact on biodiversity changed in relation to management practices for each taxonomic group. Additional analyses in the Supporting Information investigated potential differences by environments (categorized as continental and marine, or urban, terrestrial, freshwater, coastland and marine), degree of protection (yes, partially, no), or EU membership status (EU member vs. nonmember states). Chi‐squared tests computed based on 2000 Monte–Carlo simulations were also used to examine associations between certain variables. All analyses were conducted in R version 4.2.2 (R Core Team 2022), using the metapackage *tidyverse* version 2.0.0 (Wickham et al. 2019) and *lessR* version 4.2.6 (Gerbing 2021).

## 3 Results

### 3.1 Response Rate and Context‐Related Data

The survey received responses from 1928 participants who completed up to Q9, representing 72% of those who started the survey (Appendix S1: Figure S1.2) and forming *the original dataset*. Of these respondents, 88% (*n* = 1701) worked in continental environments (including freshwater and terrestrial environments), 3% (*n* = 64) focused exclusively on marine environments, and 8% (*n* = 163) worked in both continental and marine environments. Participants responding exclusively on the basis of a single environment or a subset of similar environments made up 48% of all responses (*n* = 919), distributed as follow: urban (11%, *n* = 97), terrestrial (39%, *n* = 355), freshwater (22%, *n* = 199), coastland (4%, *n* = 41), and marine (25%, *n* = 277) (Appendix S1: Figure S1.3). Based on this information, *the continental* vs. *marine dataset* contains 2091 entries, while *the specific dataset* focusing on single‐environment responses comprises 919 entries.

**FIGURE 1:**
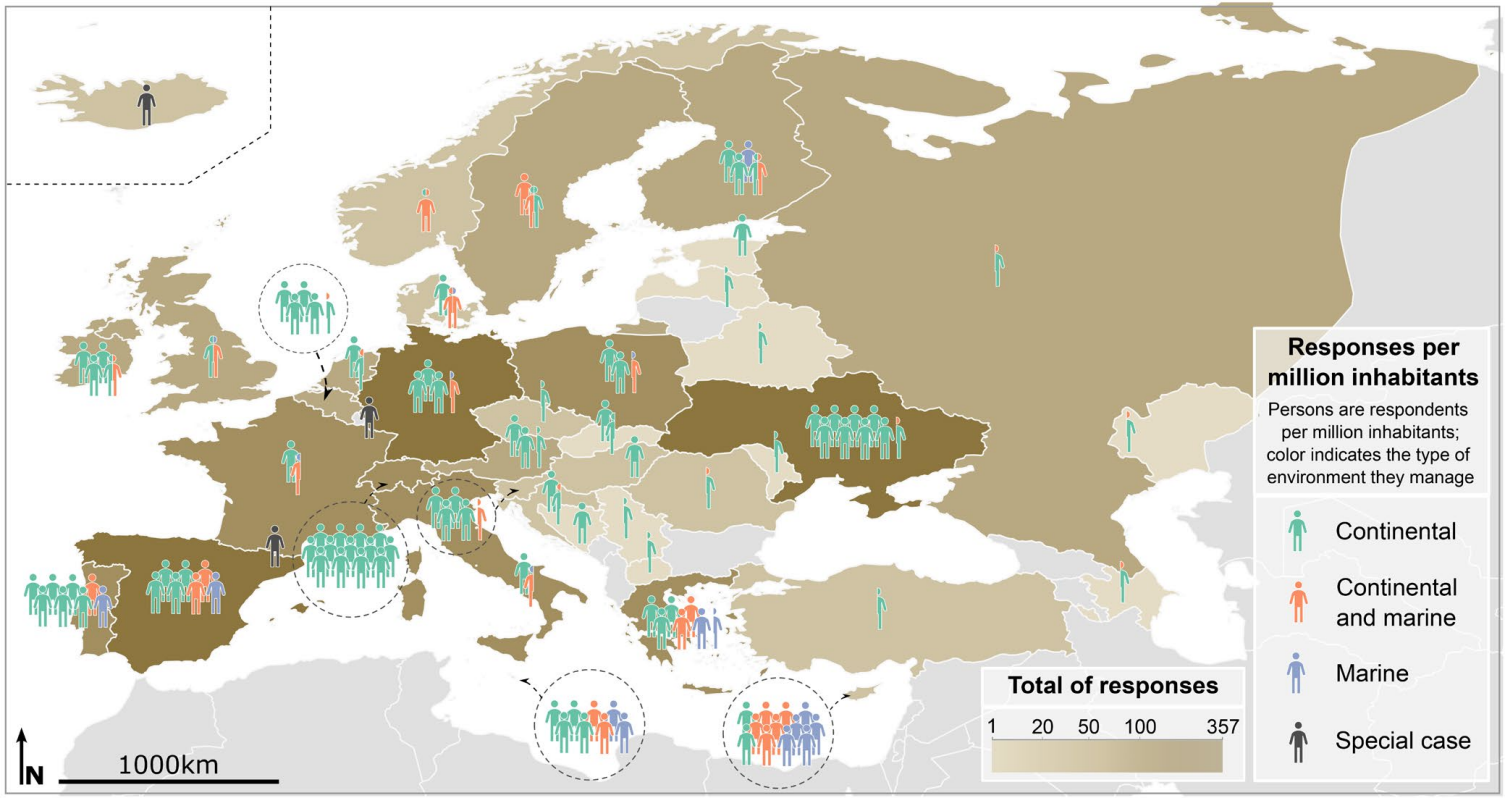
Survey responses received from each country in absolute numbers (represented by brown shades) and adjusted per million inhabitants (human icons). Each human icon signifies one respondent per one million inhabitants, with colors indicating the type of environment they manage. “Special case” refers to countries with small populations (such as Andorra, Iceland, or Liechtenstein, all with populations under 375,000, when the survey took place) where the relative number of respondents was disproportionately higher in comparison with larger countries. Detailed data can be found in Appendix S1: Table S1.4. Map lines delineate study areas and do not necessarily depict accepted national boundaries.

Responses came from 41 countries, with the majority (68%) coming from EU Member States where most collaborators dedicated efforts to contact potential respondents (Figure 1). Ukraine, Spain, and Germany had the highest absolute number of responses, while Vatican City, Andorra, Liechtenstein, and Iceland had the highest response rate per capita (Appendix S1: Table S1.4, Figure S1.4). In most countries, respondents predominantly worked in continental environments (81% ± 24%; mean ± SD), except in Sweden and Norway, where the majority worked in both continental and marine environments (> 64%). Respondents from Cyprus mainly worked in marine environments (45%) (Figure 1 and Appendix S1: Figure S1.4).

**FIGURE 2:**
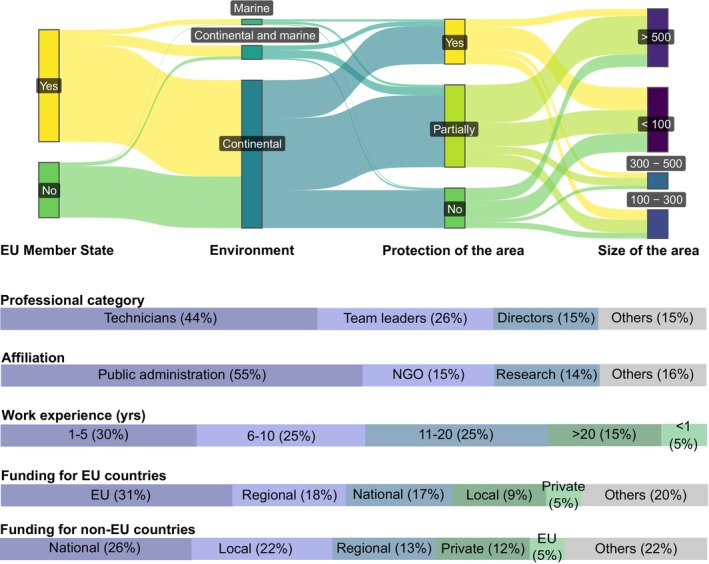
Characterization of the areas considered in the survey on the management of invasive alien species in Europe and the professional profile of the respondents. Data derived from questions Q2, Q4, Q20, Q22, Q23, Q25–Q27 of the survey (Appendix S2), using the original dataset.

Most participants worked in protected (27%) or partially protected areas (50%), which ranged in size from < 100 km^2^ (39%) to > 500 km^2^ (35%). Most respondents were professional technicians, followed by team leaders and directors. Over half (55%) of the respondents were public administration employees, followed by employees from NGOs and research institutions (~15% each). Around 50% of respondents had between 6 and 20 years of experience, while 35% were recently appointed (< 6 years) and only 15% had > 20 years of experience (Figure 2). The main source of funding for IAS management came from the public sector, both in EU and non‐EU countries. Within EU Member States, funding was predominantly sourced from the European Union, primarily through LIFE projects (52%) and the European Regional Development Funds (27%). Some non‐EU countries also received funding from the EU, possibly through transboundary projects. The key distinction in funding sources between EU and non‐EU countries was in private sector contributions, with non‐EU countries reporting a higher proportion of private funding (Figure 2).

### 3.2 Management Measures

**FIGURE 3:**
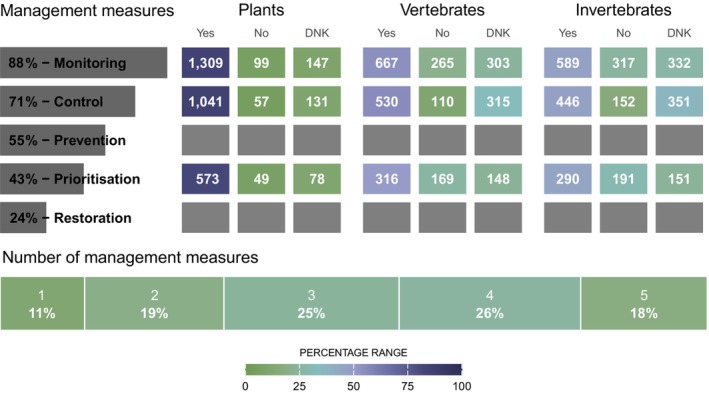
Management measures selected by respondents of the survey on the management of invasive alien species in Europe. The left bar chart shows the proportion of respondents conducting each management measure separately, while the bottom bar the proportion of respondents conducting one to five measures simultaneously within their areas (based on question Q9, Appendix S2, and using the original dataset). The heatmaps indicate the number of responses by taxonomic group for monitoring (Q11, Q12), prioritisation (Q14, Q15), and control (Q17), using the continental vs. marine dataset. Cell shading in green and blue tones represent the percentage of responses for each combination of management measure and taxonomic group. Note that the survey did not inquire about prevention and restoration measures for specific taxonomic groups, resulting in empty cells where information is missing. The abbreviation DKN refers to “Do not know” responses.

Monitoring and control were reported as the most commonly implemented measures against IAS across Europe since 2015 (Figure 3). Prevention and prioritization ranked third and fourth respectively in both EU Member States and non‐EU countries, but were slightly more important in non‐EU countries (Appendix S1: Figure S1.5A). Notably, restoration was consistently the least implemented measure. Most respondents reported that two to four management measures were implemented simultaneously (70%, Figure 3). Plants were the most frequently reported managed group of IAS, particularly in terrestrial environments (Figure 3 and Appendix S1: Figure S1.5B). The management of vertebrates received more attention in freshwater than in other environments, and more attention in EU Member States than in non‐EU countries. In contrast, invertebrates received slightly more consideration in non‐EU than in EU countries (Appendix S1: Figure S1.5A,B). However, many respondents were uncertain about measures for vertebrates and invertebrates, as evidenced by a high proportion of “Do not know” responses (Figure 3 and Appendix S1: Figure S1.5). The degree of protection of the area under consideration appears to have little influence on management efforts and the number of “Do not know” responses, although invertebrates in protected areas and vertebrates in unprotected areas are proportionally less managed than in the other areas (Appendix S1: Figure S1.5C).

Monitoring comprised both the detection of newly introduced species and the surveillance of established species (Box 1), making it challenging to distinguish between these two actions. Most respondents reported monitoring the number of species (57%) and the area occupied by IAS (52%), while fewer reported monitoring impacts (35%) (Appendix S1: Figure S1.6). Among the latter, the impact on biodiversity was the most frequently monitored impact (52%). Among taxonomic groups, plants were most frequently monitored (> 74%), while information about vertebrates and invertebrates remained largely unknown (> 26%, Appendix S1: Figure S1.6).

Most respondents reported an increase in control measures since 2015, but many were unsure about the control of invertebrate and vertebrate species, with “Do not know” responses at 44% and 37%, respectively (Appendix S1: Figure S1.7A). Regardless of the taxonomic group, 40% of respondents participated in control‐dedicated efforts to eradicate newly introduced IAS or established populations from the managed area (Appendix S1: Figure S1.7B). Complete eradication was achieved in only 5% of cases, while partial eradication in 25%. Success rates were higher in plants (8% complete and 43% partial), followed by vertebrates (5% and 16%, respectively) and invertebrates (2% and 11%, respectively) (Appendix S1: Figure S1.7C). Among those who achieved complete or partial eradication, 37% accomplished it during the early stages of invasion when the species was not yet established (i.e., rapid eradication, Appendix S1: Figure S1.7D).

Approximately half of the respondents reported the implementation of preventive measures (Figure 3), with the majority engaged in raising public awareness (93%; Appendix S1: Table S1.6). The next most frequently reported actions were guidance on eradication or control (60%), volunteer‐based removal efforts (43%), volunteer‐based early detection and reporting (41%), and promoting native species (35%). In contrast, biosecurity measures such as decontamination of vehicles and equipment were the least frequently reported actions (22%) (Appendix S1: Table S1.6). Prioritization measures (including both the evaluation of risks as well as the establishment of priorities for action) were also undertaken by a high proportion of participants (43%) (Figure 3). Among those who did report prioritization, plants were the main focus both in developing priority lists of species (75%) and priorities for invaded sites (67%) (Appendix S1: Figure S1.8), as with monitoring and control.

The MCAs examining the relationship between management measures and the environment/protection status of the areas captured > 50% of the variance with the first two dimensions (Appendix S1: Figure S1.9). Interestingly, neither the environment nor the protection status were associated with management measures. However, when considering these variables by taxonomic groups, the marine environment appeared to be slightly associated with a lack of management measures or a higher number of “Do not know” responses for plants. Similarly, vertebrates and invertebrates in marine and coastland environments exhibited a greater association with higher numbers of “Do not know” responses regarding the implementation of management measures in the area of concern. The protection status did not affect how the different taxonomic groups were managed, although vertebrates received more attention in protected areas and invertebrates did in nonprotected ones (Appendix S1: Figure S1.9).

### 3.3 IAS Trends

**FIGURE 4:**
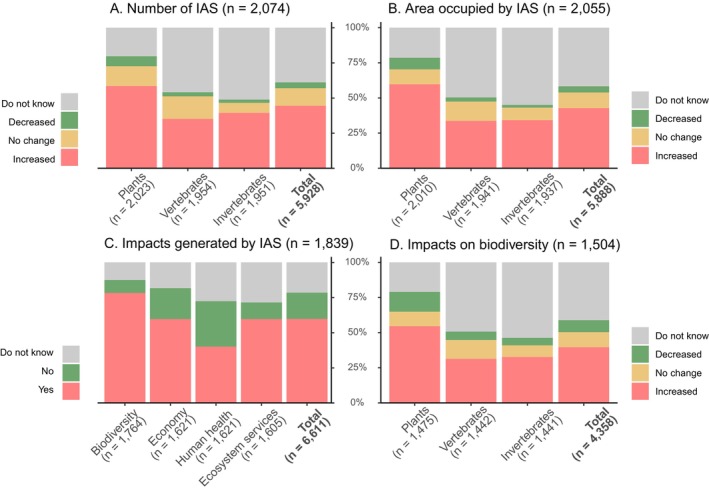
Trends in invasive alien species (IAS) across taxonomic groups in Europe. Charts show: (A) trends in the number of IAS; (B) trends in the area occupied by IAS; (C) proportion of impacts from IAS on different sectors; and (D) trends in the impact of IAS on biodiversity. The number of respondents for each question is indicated in the graphs' title, while the “n” in each column represents the responses for each taxonomic group. Data derived from questions Q5 to Q8 of the survey (Appendix S2), using the continental vs. marine dataset for panels A, B, and D and the original dataset for panel C.

Respondents indicated a perceived increase in both the number of IAS (i.e., species richness) and the area occupied by them since 2015 across all taxonomic groups (44% and 43%, respectively), with plants showing the largest increase (> 58%, Figure 4A,B). While the percentage of responses indicating a “Decrease” was low (< 8%), plants also had the highest percentage of “Decrease” responses. There is considerable uncertainty about trends in invertebrates and vertebrates, with approximately half of responses being “Do not know” (> 46%, Figure 4A,B). This uncertainty is particularly pronounced for vertebrates in non‐EU countries (> 67%), which present a proportionally higher number of “Do not know” responses compared to EU countries (< 41%) or other taxonomic groups (< 59%, Appendix S1: Figure S1.10). We found a positive association between the perceived trends of the number of IAS and the area occupied by them across taxonomic groups (Appendix S1: Figure S1.11; chi‐squared test, *p* < 0.005). Reported increases in IAS numbers typically corresponded to expanding areas (79%–92%). Given that both variables show similar statistical patterns, our analyses examining the relationship between IAS trends and management measures focus on the number of species.

Respondents noted a range of impacts associated with IAS, with impacts on biodiversity being the most frequently mentioned (72%). Responses demonstrated less certainty regarding impacts on the economy, human health, and ecosystem services (> 30%, Figure 4C). Perceived impacts were similar in EU and non‐EU countries, except for impacts on biodiversity and human health. EU Member States presented higher impacts on biodiversity (82% vs. 69%), while non‐EU countries showed higher impacts on human health (51% vs. 35%) (Appendix S1: Figure S1.10). The trends in impacts on biodiversity across taxonomic groups mirrored those observed for the number of IAS and the area they occupy. Impacts of plants were the most widely known (perceived as increasing by 55%), while respondents reported to lack information regarding the impacts of vertebrates and invertebrates (> 50%, Figure 4D). This is especially true for vertebrates in non‐EU countries, which present the highest proportion of “Do not know” responses (67%) and the lowest proportion of increases (13%). While reports of “No change” were generally low, plants, and vertebrates were the taxonomic groups with the highest proportion of such responses in impacts across EU and non‐EU countries (Appendix S1: Figure S1.10).

**FIGURE 5:**
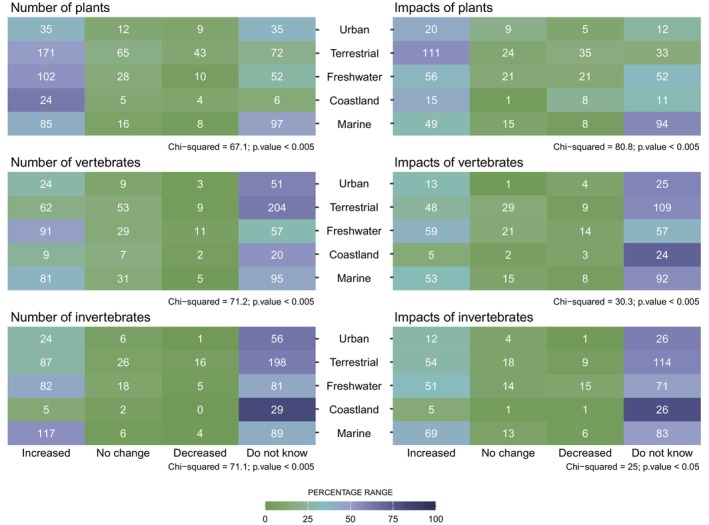
Heatmaps showing the relationship between trends in the number of invasive alien species (left panels) and their impact on biodiversity (right panels) across taxonomic groups and categories of environments. Cell values indicate the number of respondents for each taxonomic group and environment combination, while shading in green and blue tones indicating the percentage of responses for each combination. The number of species and the area they occupy are highly correlated (Appendix S1: Figure S1.11), making the results for the number of species representative of both variables. Data derived from questions Q4, Q5, and Q8 of the survey (Appendix S2), using the specific dataset. Chi‐squared and *p* values computed based on 2000 Monte–Carlo simulations.

The analysis of differences across taxonomic groups and environments regarding trends in numbers of IAS and impacts across taxonomic groups indicated a considerably high number of “Do not know” responses, except for plants in terrestrial environments (Figure 5). Plants showed substantial increases in species numbers in both terrestrial, freshwater and coastland environments, with increases in impacts particularly pronounced in terrestrial settings. Vertebrates and invertebrates increased in the number of IAS in freshwater and marine environments (Figure 5 and Appendix S1: Figure S1.12). When examining variations across taxonomic groups and degree of protection, we found that, as expected, partially protected areas experienced greater increases in the number of IAS compared with fully protected areas, regardless of the taxonomic group. Surprisingly, respondents reported greater increases in IAS numbers in both partially and fully protected areas relative to nonprotected areas. Note that the number of “Do not know” responses were high for trends in the number of vertebrate and invertebrate IAS across all levels of protection (Appendix S1: Figure S1.13).

**FIGURE 6:**
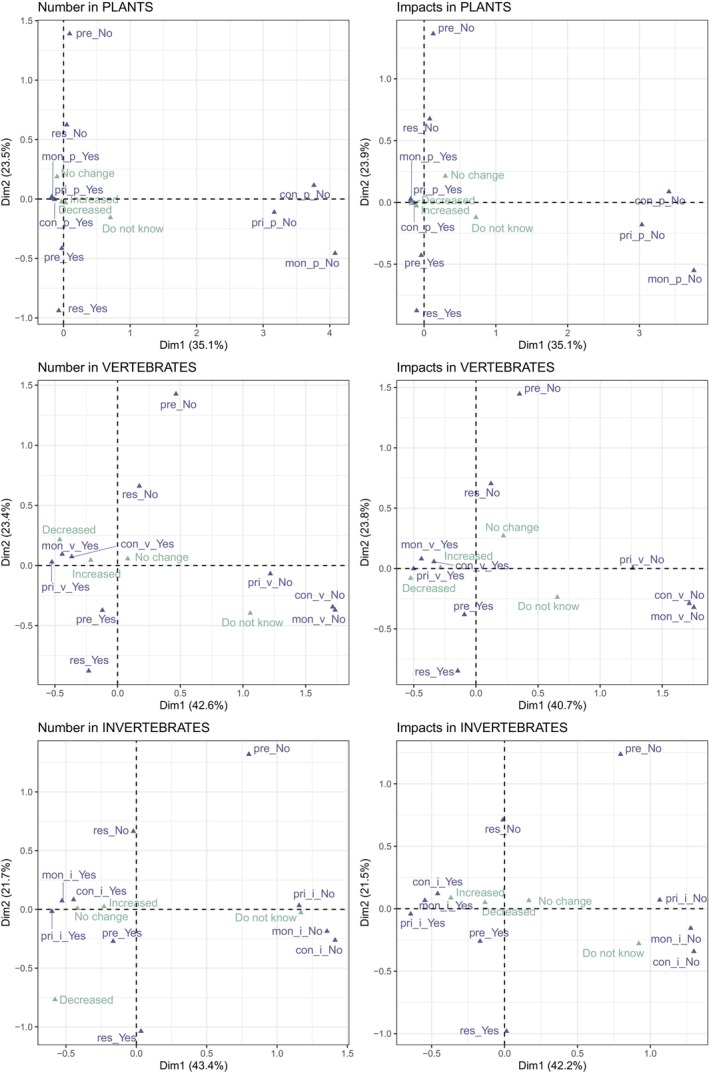
Multiple correspondence analysis (MCA) examining the relationship between management measures and trends in number of species (left panel) and impacts of invasive alien species on biodiversity (right panel) across taxonomic groups. Main variables are shown in dark purple and supplementary variables in soft green. Data derived from questions Q5 (for number of IAS), Q8 (impacts on biodiversity), Q9 (prevention and restoration), Q11–Q12 (monitoring), Q14–Q15 (prioritization), and Q17 (control) of the survey (Appendix S2), using the continental vs. marine dataset. See Appendix S1: Figure S1.14 more information on the weight of variables and the variation explained by each MCA dimension. The abbreviations refer to monitoring (mon), prevention (pre), prioritization (pri), control (con), and restoration (res).

The MCAs examining the relationships between perceived trends in IAS numbers and management measures across taxonomic groups captured > 50% of the variance with the first two dimensions (Figure 6). Dimension 1 was positively associated with responses indicating the absence of three actions: monitoring, prioritization, and control of IAS, while Dimension 2 was positively associated with responses indicating the absence of prevention and negatively associated with presence of restoration (Appendix S1: Figure S1.14). Results revealed some differences among taxonomic groups. For instance, trends in the number of invasive alien plants did not show any discernible pattern, while there is a relation between the absence of management practices (i.e., monitoring, prioritization, and control) and “Do not know” responses regarding trends in vertebrates and invertebrates. A decrease in the number of invasive alien invertebrate species was also slightly associated with the presence of restoration practices (Figure 6).

## 4 Discussion

This study captures the knowledge of local and regional managers across Europe on recent trends in the number of IAS, the area they occupy and their impacts, in addition to providing an overview of management efforts applied and their effectiveness. The high response numbers achieved in our study (*n* = 1928), relative to other surveys that have targeted IAS experts (Braun, Schindler, and Essl 2016; Dawson et al. 2016; Olszańska, Solarz, and Najberek 2016), provides a solid basis for concluding on IAS trends and management efforts at a continental scale. Overall, our findings indicate widespread implementation of various actions to mitigate biological invasions across Europe, with monitoring and control emerging as the most frequent strategies. Despite these efforts, respondents indicate that the number of IAS, the area occupied by IAS and the impacts caused by IAS have increased in recent years across all taxonomic groups, particularly in plants. However, a large proportion of participants are still uncertain, especially about trends in vertebrates and invertebrates. While the adoption of monitoring is consistent across Europe, there are some discrepancies between EU and non‐EU countries regarding the relative importance of control, prevention, and prioritization measures.

### 4.1 Management Measures

Monitoring the number of IAS and the extent of their invasion is crucial in establishing management priorities and effectively allocating the limited resources available. It provides essential information for early detection and rapid response, which can enhance the implementation of eradication measures and support the identification and control or preventive pathways (IPBES 2023; Latombe et al. 2017). Increased monitoring of IAS in EU countries could be attributed to mandatory requirements under EU IAS Regulation (European Union 2014), which requires EU Member States to establish a surveillance system to collect and record data on IAS and prioritize management measures based on the evaluation of risks and their costs effectiveness (European Commission 2021b). The high engagement of non‐EU countries in monitoring however suggests that other factors may play an important role too. While monitoring is the most employed measure across Europe, prioritization appears as the fourth most frequent measure among the five available. As prioritization of management efforts is typically conducted at higher administrative levels, this may explain why many local managers do not engage in it as much as in monitoring and control.

Control was the second most commonly used management measure. As with monitoring and prioritization, plants receive twice as much attention as vertebrates and invertebrates in control efforts. This is also reflected in the respondents' uncertainty about management measures and recent trends for vertebrates and invertebrates, highlighting a lack of knowledge for certain taxonomic groups (IPBES 2023). Interestingly, while freshwater environments show far fewer management responses compared with terrestrial ones, the management of vertebrates in this realm resembles that of plants. This may be attributed to the emphasis placed on invasive fish management (e.g., De Santis et al. 2024). Regardless of the taxonomic group and environment, the importance given to IAS across Europe is reflected in the increase in control efforts since 2015.

Among those applying control measures, few respondents intended to eradicate IAS populations from their management areas (40%) and even fewer succeeded in doing so (5% of those who tried). Similar findings were observed by the European Commission when examining management conducted on IAS of Union concern by EU Member States between 2015 and 2018 (European Commission 2021b). Half of the countries notified early detections of newly introduced species, but only 43% of them were considered to be eradicated and 25% were under eradication (Cardoso et al. 2021). Managers responding to the survey also indicated that eradication attempts often fail or are only partially successful, with success rates for plants being higher than those for vertebrates and invertebrates. Interestingly, this occurs despite the general belief that plants are generally more challenging to eradicate than animals (Pluess et al. 2012a, 2012b; Shackleton et al. 2022). Early stages or isolated patches of plant invasions require continued management efforts to achieve a complete removal, but managing vertebrates and invertebrates presents considerable difficulties due to their high mobility and stronger public opposition (Robertson et al. 2017).

Preventing the introduction and expansion of IAS into neighboring areas is recognized as the most effective measure to mitigate potential socioecological impacts. The costs of preinvasion management are up to 25 times lower than of postinvasion management (Cuthbert et al. 2022). However, only half of the surveyed managers adopted prevention measures, with a slightly higher prevalence in non‐EU countries compared to EU ones. Raising public awareness appears to be the most widely adopted action, followed by guidance on eradication or control of IAS, aligning with the European Commission reports covering the period from 2015 to 2018 (European Commission 2021b). Most efforts consist in the creation of dedicated websites and smartphone applications, as well as awareness campaigns and guides for the identification of species and pathways of introduction (European Commission 2021b). These results corroborate the idea that local and regional managers focus more on responding to invasions than working on prevention (Pyšek et al. 2013). Proactive management measures are expected to be conducted at the country level or across several countries, as required by the EU IAS Regulation (European Union 2014) and the European and Mediterranean Plant Protection Organisation (Branquart et al. 2016).

The restoration of invaded areas is less frequently reported, yet it is recognized as a measure for enhancing the resilience of recipient ecosystems against invasions (D'Antonio, August‐Schmidt, and Fernandez‐Going 2016). Numerous studies have shown that integrating eradication and control measures into broader ecosystem restoration efforts, rather than implementing them in isolation, leads to greater effectiveness (Zavaleta, Hobbs, and Mooney 2001). Relevant EU policy and legislation (such as the Nature Protection Directives, the Water Framework Directive and the Nature Restoration Law) also imply the use of restoration measures for habitat conservation. More specifically, the EU IAS Regulation has a provision (Article 20) on the restoration of ecosystems affected by IAS to recover from their effects. The results of our survey may indicate that this important provision is not receiving appropriate attention by Member States, perhaps because proper guidance on how to do this is lacking or economic limitations (Ayres et al. 2014). Despite this, several conservation projects in Europe are addressing restoration of invaded ecosystems (e.g., LIFE DUNIAS, LIFE20 NAT/BE/001442, https://www.natuurenbos.be/projecten/life‐dunias; or LIFE RESILIAS, LIFE19 NAT/NL/000821; https://www.resilias.eu). In fact, we argue that in many areas, restoration is often the primary objective of actions taken against IAS and therefore closer to the reality of conservation action, especially in protected areas, than systematic eradication or control of specific IAS. Furthermore, the lack of reported restoration efforts in our survey may indicate a disconnection between the invasion and ecological restoration communities, suggesting that closer integration of these disciplines might advance both fields.

### 4.2 IAS Trends

Despite management measures concerning biological invasions, the number of IAS, the area they occupy, and their impacts on biodiversity are still increasing in Europe, according to the local and regional managers we surveyed. This is consistent with known data on introduction rates and impact assessments (IPBES 2023; Kumschick et al. 2015; Seebens et al. 2021, 2017). Particularly increasing trends were reported for IAS plants, despite also reporting higher investments in management (i.e., monitoring, prioritization and control) compared to other taxonomic groups. Respondents were generally less confident in assessing trends for IAS of invertebrates and vertebrates, particularly in non‐EU countries, though they reported the highest increases in freshwater and marine ecosystems (e.g., Anastácio et al. 2019; Chainho et al. 2015). This may reflect both a lack of knowledge or data on these groups and environments, and the inherent vulnerability of these ecosystems to invasions. These findings highlight the need for context‐specific approaches that account for the unique conditions of these environments (Mathers, Guareschi, and Pattison 2022).

There are some notable discrepancies between our results derived from managers' knowledge and those reported by the European Commission on the distribution of IAS of Union concern between 2015 to 2018 (European Commission 2021b). While “No change” rates (~12%) are similar between the official data and our survey (14% reported by the European Commission vs. 12% for our survey), our survey reported consistently higher rates for increase in the number of IAS (17% vs. 44%) and lower rates for decrease (21% vs. 4%) (Figure 4). One possible explanation for this discrepancy is the difference in methodologies between the two studies. Our survey asked about changes in all IAS and separated them by taxonomic groups, while the European Commission focused on the species included in the list of Union Concern. We also asked about perceptions of increase or decrease in IAS numbers, area, and impacts across managers' areas in Europe, a method that differs from the Commission's more targeted approach on specific IAS populations or the total populations across EU Member States. Additionally, the respondents to the survey and those who report to the European Commission are not always the same and may have different knowledge associated with different scales and contexts.

It is important to note that the trends in our study are primarily based on the number of IAS (i.e., species richness) rather than the area occupied by them. The positive correlation found between species number and area makes sense in two ways: a larger area might be occupied if an already established population expands, or if new species establishe in areas that were previously not occupied by any IAS. These are two different processes, although both can happen simultaneously within a given area (Blackburn, Cassey, and Pyšek 2021). However, a positive correlation, as observed in our study, might not have a causative explanation if the number of IAS and the area occupied by IAS are increasing independently (e.g., if a larger occupied area is mainly due to population expansion of only a few already established species). Overall, it is important to highlight that by asking about both the number of IAS (i.e., species richness) and the area occupied by IAS, we are hopefully capturing trends in both species' number and population expansion of already established species.

With regard to impacts, effects of IAS on biodiversity are the ones most reported, while the impacts on the economy, human health, and ecosystem services are less certain (Bacher et al. 2023). There is a noticeable lack of certainty regarding the impacts of invertebrates and vertebrates, coupled with notable disparities between EU and non‐EU Member States. EU Member States show greater certainty in quantifying the impact of IAS than non‐EU Member States, with vertebrates and invertebrates presenting the highest divergences. An impact assessment of IAS in Europe revealed many environmental and socioeconomic impacts across different taxonomic groups such as fish, plants, and arthropods (Kumschick et al. 2015). The assessment interestingly noted that while fish and plants generally have more documented environmental impacts, arthropods tend to have more pronounced socioeconomic impacts. Furthermore, in mammals and birds, those with lower socioeconomic impacts inflict greater environmental damage, and vice versa.

Despite the significant and increasing investments and resources dedicated to IAS management globally (Moodley et al. 2022) and at the European level since the 1990s (Scalera 2010; Scalera et al. 2017), our findings suggest that management efforts conducted at regional and local scales are not sufficient to reduce the presence and impacts of IAS (Monaco and Genovesi 2014). The absence of a clear relationship between management measures and the trends in IAS does not necessarily indicate ineffective local/regional management, for example if the rate of introductions greatly exceeds the management capacities (Tu and Robinson 2013). It could be the situation of areas with some degree of environmental protection, where respondents reported an increase in both the number of IAS and the areas they occupy, despite having more resources available for management.

The smaller reported increase in unprotected areas compared to protected ones could reflect a lack of reliable information on IAS in these regions. Protected areas typically receive more attention in conservation and management planning, leading to more resources being allocated for IAS control and monitoring (Moodley et al. 2022). In this regard, the less pronounced increase in protected areas compared to partially protected areas may indicate that better protection of natural areas is always a good strategy to reduce invasion pressure. Areas with a protected status (e.g., legal protection as a nature reserve, national park) may be more resistant or resilient to invasions, for instance because the presence of intact native vegetation hampers alien species establishment or because some nature reserves are less accessible to visitors that can bring in propagules (Foxcroft et al. 2013; Lonsdale 1999; Pys̆ek, Jaros̆ík, and Kuc̆era 2002). However, we did not conduct an analysis of the characteristics of these areas (such as level of protection, number of visitors, size, and degree of invasions), which could influence these results.

The dynamics of IAS are complex and influenced by a range of factors operating at both global and local scales, including considerable time lags (Courchamp et al. 2017; Dana et al. 2019; Essl et al. 2015; Piria et al. 2017). Management efforts are often reactive, focusing primarily on areas where IAS are already established rather than adopting anticipatory or preventive measures, such as addressing incipient IAS or uninvaded areas (Roy et al. 2019; Thorpe et al. 2014); for a more general context, Weise et al. (2020) could help generating a positive relationship between management intensity and IAS trends. These management‐related factors, along with data limitations such as varying response rates across regions or the profile of the targeted audience, can influence the interpretation of the survey results and the assessment of management effectiveness. While these factors should be considered when interpreting the data, the high response rate achieved in this study underscores the robustness of the findings and their importance.

## 5 Conclusions

This study is the first of its kind to provide an overview of managers' opinion on IAS management at local and regional scales across Europe and its effects on biological invasions levels across environments and taxonomic groups. It highlights how the knowledge of managers and practitioners is an essential asset for capturing and expanding our understand of IAS trends and management. Respondents consistently reported increasing trends in the number, area occupied, and impact of IAS, despite ongoing management efforts. This highlights that current practices are insufficient to achieve international biodiversity objectives such as those outlined in Target 6 of the Kunming‐Montreal Global Biodiversity Framework. Local management efforts alone cannot address this global and interconnected problem adequately, so it is crucial to foster vertical (between on‐the‐ground managers and policymakers) and horizontal (between countries) cooperation across Europe. Combined with continued dedication to refining monitoring methodologies and data reporting, these efforts will enable us to progress toward the Framework's ambitious target of reducing IAS levels by 2030 (CBD 2022).

## Author Contributions

**Carla Garcia‐Lozano:** conceptualization, data curation, formal analysis, investigation, methodology, project administration, software, validation, visualization, writing – original draft, writing – review and editing. **Josep Pueyo‐Ros:** software. **Quim Canelles:** formal analysis, writing – review and editing. **Guillaume Latombe:** formal analysis, writing – review and editing. **Tim Adriaens:** investigation, writing – review and editing. **Sven Bacher:** investigation, writing – review and editing. **Ana Cristina Cardoso:** investigation, writing – review and editing. **Michelle Cleary:** investigation, writing – review and editing. **Lluís Coromina:** validation, writing – review and editing. **Franck Courchamp:** investigation, writing – review and editing. **Wayne Dawson:** investigation, writing – review and editing. **Maarten de Groot:** investigation, writing – review and editing. **Franz Essl:** investigation, writing – review and editing. **Belinda Gallardo:** investigation, writing – review and editing. **Marina Golivets:** investigation, writing – review and editing. **Erja Huusela:** investigation, writing – review and editing. **Miia Jauni:** investigation, writing – review and editing. **Sven D. Jelaska:** investigation, writing – review and editing. **Jonathan M. Jeschke:** investigation, writing – review and editing. **Stelios Katsanevakis:** investigation, writing – review and editing. **Melina Kourantidou:** investigation, writing – review and editing. **Ingolf Kühn:** investigation, writing – review and editing. **Bernd Lenzner:** investigation, writing – review and editing. **Brian Leung:** conceptualization, writing – review and editing. **Elizabete Marchante:** investigation, writing – review and editing. **Colette O'Flynn:** investigation, writing – review and editing. **Cristian Pérez‐Granados:** investigation, writing – review and editing. **Jan Pergl:** investigation, writing – review and editing. **Pavel Pipek:** investigation, writing – review and editing. **Cristina Preda:** investigation, writing – review and editing. **Filipe Ribeiro:** investigation, writing – review and editing. **Helen Roy:** investigation, writing – review and editing. **Riccardo Scalera:** investigation, writing – review and editing. **Menja von Schmalensee:** investigation, writing – review and editing. **Hanno Seebens:** investigation, writing – review and editing. **Róbert A. Stefánsson:** investigation, writing – review and editing. **Barbara Tokarska‐Guzik:** investigation, writing – review and editing. **Elena Tricarico:** investigation, writing – review and editing. **Sonia Vanderhoeven:** investigation, writing – review and editing. **Vigdis Vandvik:** investigation, writing – review and editing. **Montserrat Vilà:** investigation, writing – review and editing. **Núria Roura‐Pascual:** conceptualization, funding acquisition, investigation, methodology, project administration, resources, supervision, validation, writing – original draft, writing – review and editing.

## Conflicts of Interest

The authors declare no conflicts of interest.

## Supporting information


Appendix S1.

Appendix S2.

## Data Availability

The data and the code that support the findings of this study are openly available in Zenodo at https://doi.org/10.5281/zenodo.14608866. Additionally, data visualizations are available through an interactive web map at https://www.nrourapascual.com/ias‐europe/.
