# Supplementary material for: Management Measures and Trends of Biological Invasions in Europe: A Survey‐Based Assessment of Local Managers
Source: Glob Chang Biol. 2025 Jan 17;31(1):e70028. doi: 10.1111/gcb.70028 (PMC11742469; doi:10.1111/gcb.70028)
Supplement: Supplementary file 1 — Appendix S1. [file GCB-31-e70028-s002.pdf]

This material serves as supplementary content for the research article titled "*Management Measures and Trends of Biological Invasions in Europe: A Survey-Based Assessment of Local Managers*" (DOI: 10.1111/gcb.70028).

Supplementary material 1

TABLES

Table S1.1
Contributors to the design and dissemination of the survey on the management of invasive alien species in Europe (available in Supplementary Material 2).

Id	Country	Design	Dissemination	Collaborators	Affiliation
1	Austria	Yes	Yes	Essl, Franz	University of Vienna, Austria
2		Yes	Yes	Lenzner, Bernd	University of Vienna, Austria
3	Andorra		Yes	Puigdemassa-Martí, Elena	University of Girona, Spain
4	Belgium		Yes	Adriaens, Tim	Research Institute for Nature and Forest (INBO), Belgium
5			Yes	Vanderhoeven, Sonia	Belgian Biodiversity Platform, Belgium
6	Czech Republic		Yes	Pipek, Pavel	Faculty of Science, Charles University, Czech Republic; Institute of Botany, Czech Academy of Sciences, Czech Republic
7			Yes	Pergl, Jan	Institute of Botany, Czech Academy of Sciences, the Czech Republic
8	Croatia		Yes	Jelaska, Sven	Faculty of Science, University of Zagreb, Croatia
9	Denmark		Yes	Kourantidou, Melina	University of Southern Denmark, Denmark
10	Finland		Yes	Jauni, Miia	Natural Resources Institute Finland (Luke), Finland
11			Yes	Huusela, Erja	Natural Resources Institute Finland (Luke), Finland
12	France	Yes	Yes	Courchamp, Franck	University Paris Saclay & Centre National de la Recherche Scientifique, France
13	Germany	Yes	Yes	Jeschke, Jonathan	Freie Universität Berlin & Leibniz Institute of Freshwater Ecology and Inland Fisheries (IGB), Germany
14		Yes	Yes	Seebens, Hanno	Senckenberg Biodiversity and Climate Research Centre, Germany
15		Yes		Kühn, Ingolf	Helmholtz Centre for Environmental Research, Germany
16		Yes		Saul, Wolf-Christian	Freie Universität Berlin & Institute for Freshwater Ecology and Inland Fisheries, Germany
17	Greece		Yes	Katsanevakis, Stelios	University of the Aegean, Greece
18	Iceland		Yes	von Schmalensee, Menja	West Iceland Nature Research Centre, Iceland
19			Yes	Stefánsson, Róbert A.	West Iceland Nature Research Centre, Iceland
20	Ireland		Yes	O'Flynn, Colette	National Biodiversity Data Centre, Ireland
21	Italy		Yes	Tricarico, Elena	University of Florence, Italy
22	Norway		Yes	Vandvik, Vigdis	University of Bergen, Norway
23	Poland		Yes	Tokarska-Guzik, Barbara	University of Silesia in Katowice, Poland
24	Portugal		Yes	Marchante, Beta	University of Coimbra, Portugal Marine and Environmental Sciences, Portugal
25			Yes	Ribeiro, Filipe	Centre (MARE), Portugal
26	Romania		Yes	Preda, Cristina	Ovidius University of Constanța, Romania
27	Slovenia		Yes	De Groot, Maarten	Slovenian Forestry Institute, Slovenia
28	Spain	Yes	Yes	Garcia-Lozano, Carla	University of Girona, Spain
29		Yes	Yes	Dana, Elías	Junta de Andalucía, Spain
30		Yes	Yes	Gallardo, Belinda	Pyrenean Institute of Ecology (CSIC), Spain
31		Yes	Yes	Roura-Pascual, Núria	University of Girona, Spain
32		Yes		Coromina, Lluís	University of Girona, Spain
33		Yes		Vilà, Montserrat	Estación Biológica de Doñana, Spain

Id	Country	Design	Dissemination	Collaborators	Affiliation
34		Yes	Yes	Pérez-Granados, Cristian	Alicante University, Spain
35	Sweden	Yes	Yes	Cleary, Michelle	Swedish University of Agricultural Sciences, Sweden
36	Switzerland	Yes	Yes	Bacher, Sven	University of Fribourg, Switzerland
37	Netherlands		Yes	van Dijk, Chris	Wageningen University & Research, the Netherlands
38	United Kingdom		Yes	Dawson, Wayne	Durham University, United Kingdom
39		Yes		Latombe, Guillaume	University of Edinburgh, United Kingdom
40		Yes		Roy, Helen	Centre of Ecology and Hydrology, United Kingdom
41	Ukraine	Yes	Yes	Golivets, Marina	Helmholtz Centre for Environmental Research, Germany
42	Across Europe	Yes	Yes	Cardoso, Ana C.	Joint Research Centre of the European Commission, Italy
43			Yes	Pastor, Teresa	Coordinator of Fedenatur in EUROPARC Europe, Spain
44		Yes	Yes	Scalera, Riccardo	IUCN/SSC Invasive Species Specialist Group, Rome, Italy
45	Other	Yes		Leung, Brian	McGill University, Canada

Table S1.2
Structure of the survey on the management of invasive alien species (abbreviated IAS) in Europe. The survey comprises 27 questions divided into two main categories (context vs. management-related questions), and each category is further subdivided into separate subcategories. The type of question (single or multiple answers, lists, tables, drag and drop, or free text), their dependency on the environment (as described in Panel S1.1), and the potential responses appear also indicated in columns. Questions have an identifier (ID), which can be used to find a specific question in Supplementary Material 2. Mandatory questions appear indicated with (*).

Categories	Subcategories	Question	Type of question	Environment	Responses	Question ID
Context-related questions	Filter local managers	Are you involved in the management of invasive alien species?*	Single answer vertical		List of options (Yes/No)	Q1
	Geographical region	Select the country of the area you manage.*	Single answer dropdown		List of options (European countries)	Q2
		Select the region of the area you manage.*	Single answer dropdown		List of options (Regions of each country)	Q3
	Environment	Select the environments that characterize your management area most accurately.*	Multiple answers vertical		List of options	Q4
	Funding	Select the funding sources you have received for the management of IAS in your area since 2015?	Multiple answers vertical		List of options	Q20
	Area characteristics	What is the size of the area you manage?	Single answer vertical		List of options	Q22
		Is your management area protected?	Single answer vertical		List of options (Yes / No / Partially / Do not know / Not applicable)	Q23
		Specify the protection figure of the area you manage.	Free text			Q24
	Professional characteristics	Select your affiliation.	Single answer vertical		List of options	Q25
		Select your professional category.	Single answer vertical		List of options	Q26
		Select the number of years you have been working on IAS management.	Single answer vertical		List of options	Q27
Management-related questions	Trends	How has the number of IAS changed?	Single answer table	Yes	List of options (Decreased / Increased / No change) by taxonomic groups	Q5
		How has the area occupied by the IAS changed?	Single answer table	Yes	List of options (Decreased / Increased / No change) by taxonomic groups	Q6
		Has any negative impact caused by IAS been detected?	Single answer table	Yes	List of options (Yes/No) by impacts	Q7
		How have the (negative) impacts on the biodiversity caused by IAS changed?	Single answer table	Yes	List of options (Decreased / Increased / No	Q8

Categories	Subcategories	Question	Type of question	Environment	Responses	Question ID
					change) by taxonomic groups	
	Practices	Which management measures have been implemented?*	Multiple answers vertical		List of options (Monitor / Prioritisation / Prevention / Control / Restoration)	Q9
		Drag and drop the previous management measures according to the time dedicated to them (1 being more dedication and 5 less dedication)	Drag and drop		List of options	Q10
		Has the number of IAS been monitored? [if monitoring is selected]	Single answer table	Yes	List of options (Yes / No / Do not know / Not applicable) by taxonomic groups	Q11
		Has the area occupied by IAS been monitored? [if monitoring is selected]	Single answer table	Yes	List of options (Yes / No / Do not know / Not applicable) by taxonomic groups	Q12
		Have the impacts caused by IAS been monitored? [if monitoring is selected]	Single answer table	Yes	List of options (Yes / No / Do not know / Not applicable) by impacts	Q13
		Has a priority list of IAS been established? [if prioritisation is selected]	Single answer table	Yes	List of options (Yes / No / Do not know / Not applicable) by taxonomic groups	Q14
		Have priorities for managing the different invaded sites been established? [if prioritisation is selected]	Single answer table	Yes	List of options (Yes / No / Do not know / Not applicable) by taxonomic groups	Q15
		Which measures have been applied to prevent the introduction or spread of IAS? [if prevention is selected]	Multiple answers vertical		List of options	Q16
		How have the actions aimed to control IAS changed? [if eradication and control are selected]	Single answer table	Yes	List of options (There have not been any / Decreased / No change / Increased) by taxonomic groups	Q17
		Has any IAS been eradicated? [if eradication and control are selected]	Multiple answers table	Yes	List of options (Yes / No / Partially / Do not know / Not applicable) by taxonomic groups	Q18
		Has any eradication been done before the establishment of populations? [if eradication and control are selected]	Single answer table	Yes	List of options (Yes / No / Do not know / Not applicable) by taxonomic groups	Q19
	Other	Use this space to share any additional information that you consider relevant about IAS management.	Free text			Q21

Table S1.3
Distribution lists used for the online dissemination of the survey to their subscribers.

Organization	Reach
IUCN SSC Invasive Species Specialist Group (https://www.iucn.org/our-union/commissions/group/iucn-ssc-invasive-species-specialist-group)	Global
The Group on Earth Observations Biodiversity Observation Network (GEO BON) (https://geobon.org/)	Global
COST Action Alien CSI (https://www.ceh.ac.uk/our-science/projects/alien-csi)	European
The beach and dune network (https://www.eucc-d.de/beach-and-dune-network.html)	European
EUROPARC Federation (https://www.europarc.org/)	EU Member States
European Alien Species Information Network (EASIN) of the Joint Research Centre (JRC) of the European Commission (https://easin.jrc.ec.europa.eu/easin)	EU Member States
Marine Strategy Framework Directive non-indigenous species expert network	EU Member States
Water Framework Directive Ecological Status Working Group	EU Member States
East and South European Network for Invasive Alien Species (ESENIAS) (https://www.esenias.org/)	Eastern and Southern Europe
Belgian Forum on Invasive Alien Species (BFIS) (https://ias.biodiversity.be/)	Belgium
ExotenNET (https://exotennet.be/)	Belgium
Rede InvECO - Portuguese Network for the Study and Management of Invasive Species	Portugal

Table S1.4

Ranking of countries based on the number of responses to the survey (n=1,928). Data are presented in (a) absolute numbers of responses, and (b) normalised responses per million inhabitants according to Eurostat, https://ec.europa.eu/eurostat, in 2022. (*) indicates EU Member States, while (°) countries where the contributors made an effort to disseminate the survey (see Table S1.1).

**Country**	**Responses (a)**	**Country**	**Responses/inhabitants (b)**
Ukraine°	357	Vatican City	1212.12
Spain*°	334	Andorra°	131.27
Germany*°	295	Liechtenstein	76.81
France*°	116	Iceland°	54.23
Switzerland°	114	Switzerland°	13.15
Poland*°	91	Cyprus*	12.28
Italy*°	90	Ukraine°	8.62
Portugal*°	85	Portugal*°	8.25
Greece*°	70	Malta*	7.75
Belgium*°	50	Spain*°	7.05
United Kingdom°	50	Greece*°	6.56
Netherlands*°	27	Belgium*°	4.33
Austria*°	24	Slovenia*°	4.27
Finland*°	22	Ireland*°	4.19
Sweden*°	22	Finland*°	3.98
Ireland*°	21	Germany*°	3.55
Russian Federation	21	Austria*°	2.69
Iceland°	20	Poland*°	2.40
Hungary*	13	Sweden*°	2.12
Denmark*°	12	Denmark*°	2.05
Cyprus*	11	France*°	1.71
Turkey	11	Netherlands*°	1.54
Andorra°	10	Italy*°	1.52
Slovenia*°	9	Croatia*°	1.49
Czech Republic*°	8	Hungary*	1.34
Croatia*°	6	Norway°	1.11
Norway°	6	Bosnia and Herzegovina	0.78
Romania*°	6	Estonia*	0.75
Malta*	4	Czech Republic*°	0.75
Azerbaijan	3	United Kingdom°	0.75
Bosnia and Herzegovina	3	Slovakia*	0.55
Liechtenstein	3	Latvia*	0.53
Serbia	3	Northern Macedonia	0.48
Slovakia*	3	Serbia	0.44
Kazakhstan	2	Moldova	0.38
Belarus	1	Romania*°	0.31
Estonia*	1	Azerbaijan	0.30
Latvia*	1	Russian Federation	0.15
Moldova	1	Turkey	0.13
Northern Macedonia	1	Belarus	0.11
Vatican City	1	Kazakhstan	0.10

Table S1.5
Combination of management measures selected by survey respondents (derived from question Q9 of the survey, Supplementary Material 2), together with the number of responses and the percentage they represent in the original dataset (n=1,928). Respondents could select up to five management measures from a list of options that included monitoring, prevention, prioritisation, control, and restoration (Box 1).

Management measures	Responses	%
monitoring & prioritisation & prevention & control	281	14.6
monitoring	230	11.9
monitoring & prevention & control	225	11.7
monitoring & prioritisation & prevention & control & restoration	218	11.3
monitoring & control	215	11.2
monitoring & prevention	108	5.6
monitoring & prioritisation & control	106	5.5
control	69	3.6
monitoring & prioritisation	68	3.5
monitoring & control & restoration	66	3.4
monitoring & prioritisation & prevention	55	2.9
monitoring & prevention & control & restoration	48	2.5
prevention	38	2.0
prevention & control	36	1.9
monitoring & prioritisation & control & restoration	35	1.8
control & restoration	33	1.7
monitoring & restoration	13	0.7
prioritisation & prevention & control	13	0.7
prioritisation & control	11	0.6
prioritisation & prevention	9	0.5
restoration	9	0.5
monitoring & prevention & restoration	7	0.4
monitoring & prioritisation & prevention & restoration	7	0.4
prioritisation	7	0.4
monitoring & prioritisation & restoration	6	0.3
prevention & control & restoration	5	0.3
prioritisation & control & restoration	5	0.3
prevention & restoration	4	0.2
prioritisation & prevention & restoration	1	0.1

Table S1.6

Prevention measures selected by survey respondents (derived from question Q16 of the survey, Supplementary Material 2), together with the number of responses and the percentage of respondents who reported applying preventive measures (n=976) in the original dataset. Respondents could select up to six prevention measures and/or add new ones. The abbreviation IAS refers to invasive alien species.

**Prevention measures**	**Responses**	**%**
Raising public awareness	905	93
Guidance on eradication or control of IAS	584	60
Volunteer-based removal efforts	419	43
Volunteer-based early detection and reporting	396	41
Promete non-invasive species	346	35
Decontamination of vehicles and/or equipment	214	22
Other	100	10

FIGURES

Figure S1.1
Number of survey responses by European regions. Respondents had to select their country and then indicate the region where the management takes place. For EU Member States, regions were defined according to the Nomenclature of Territorial Units for Statistics Level 2 (NUTS 2). For non-EU Member States, regions with similar administrative levels were selected. Map lines delineate study areas and do not necessarily depict accepted national boundaries.

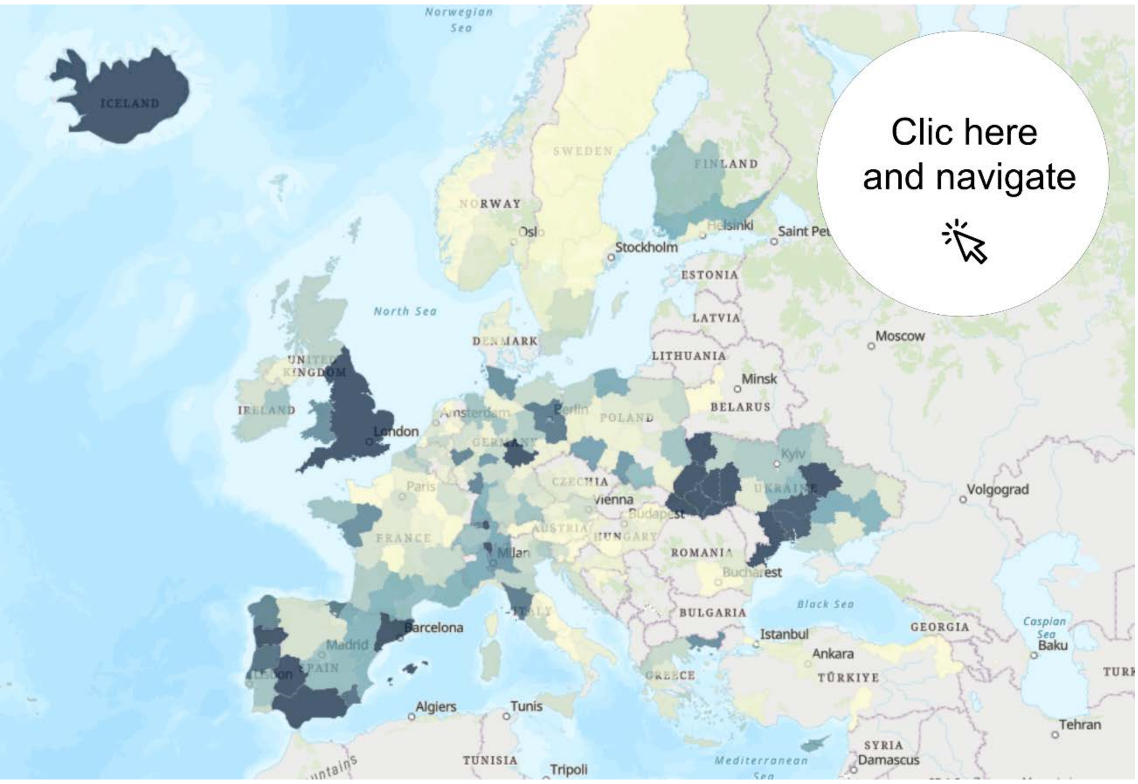

Click on the figure to access the interactive map

Figure S1.2
Percentage of respondents across the survey questions, with each question identified by its ID and grouped into categories and subcategories (Table S1.2, Supplementary Material 2). The number of respondents that initiated the survey was 2,662.

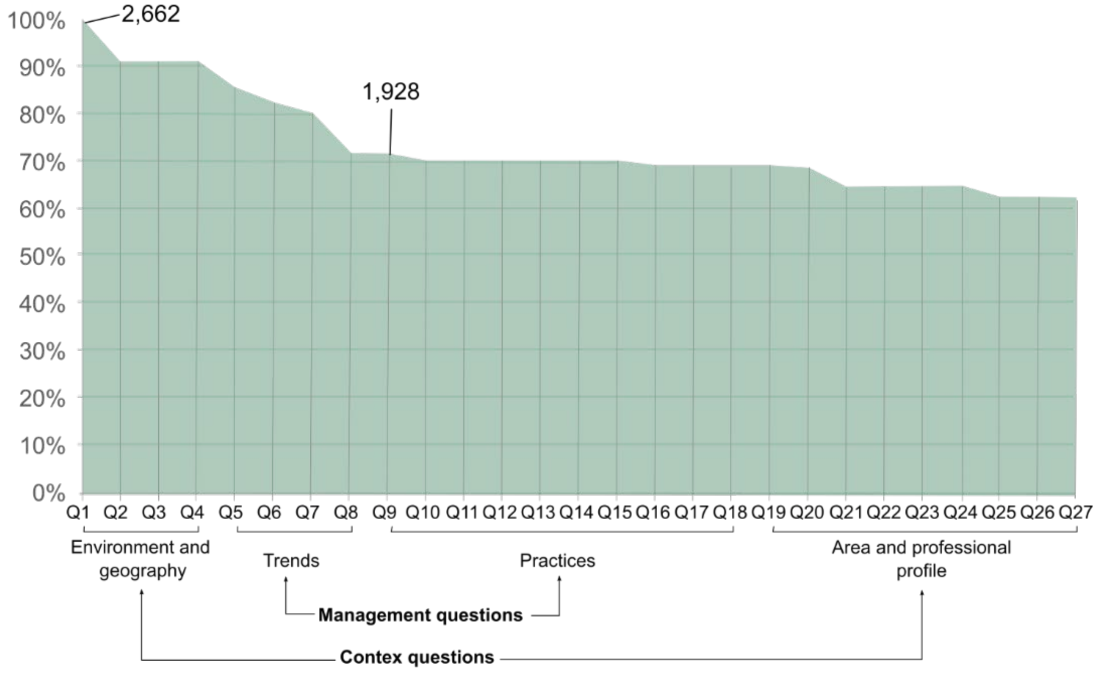

Figure S1.3
Number of survey responses categorised by type of environment: continental (1,701 responses), marine (64 responses; abbreviated M), or both (163 responses). Continental responses are further categorised by specific groups of environments: urban (U), terrestrial (T), freshwater (F), and coastland (C). See Panel S1.1 for more details on the calculation.

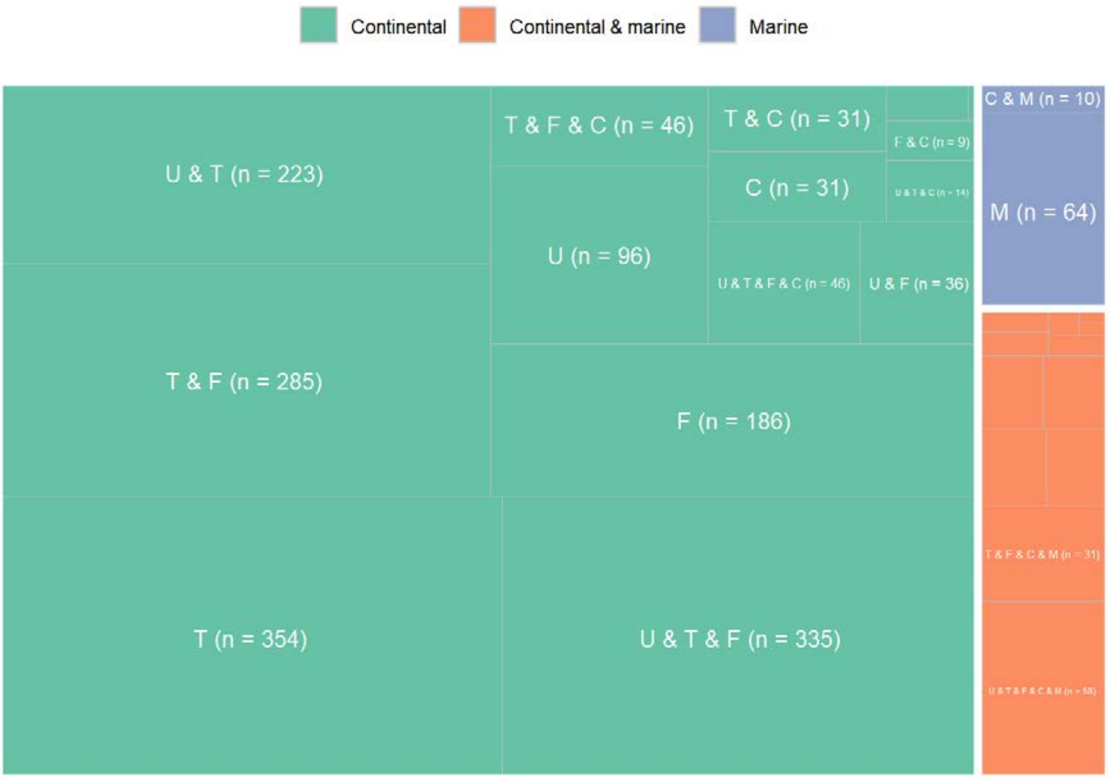

Figure S1.4
Number of survey responses per country, categorised by the type of environment: continental, marine, or both.

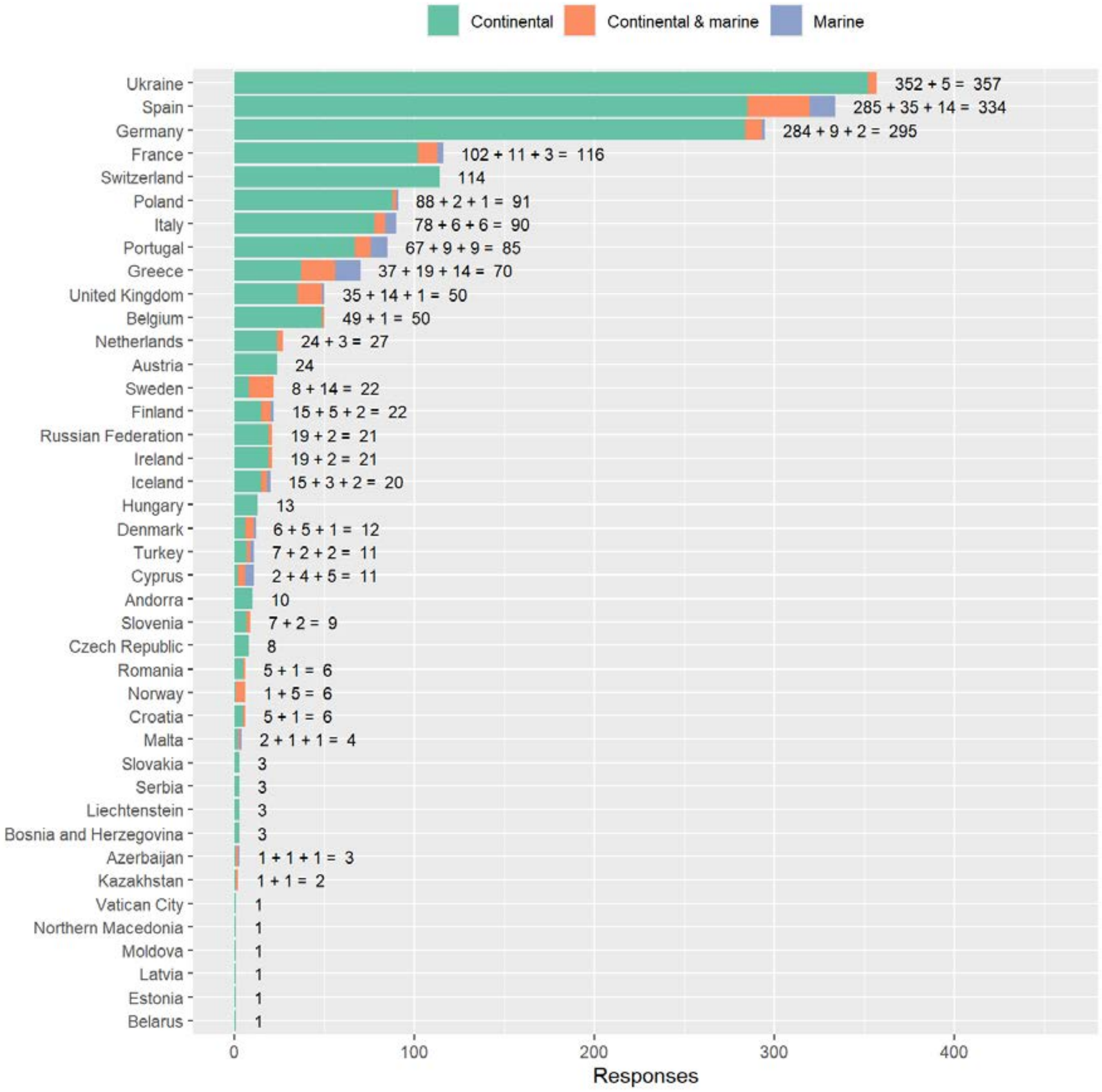

Figure S1.5
Management measures selected by survey respondents, separated according to: (A) European Union membership (EU or non-EU Member States); (B) the environments being managed (marine, coastland, freshwater, terrestrial, urban); and (C) the level of protection of the area (protected, partially protected, unprotected). The left bar chart shows the proportion of respondents conducting each management measure separately (based on question Q9 of the survey, Supplementary material 2) and the right heatmaps the number of responses by taxonomic group for monitoring (Q11, Q12), prioritisation (Q14, Q15), and control (Q17), using the continental vs. marine dataset. Cell shading in green and blue tones represent the percentage of responses for each combination of management measure and taxonomic group. Note that the survey did not inquire about prevention and restoration measures for specific taxonomic groups, resulting in empty cells where information is missing. The abbreviation DKN refers to "Do not know" responses.

A. European Union membership
EU Member States (n = 1,451)

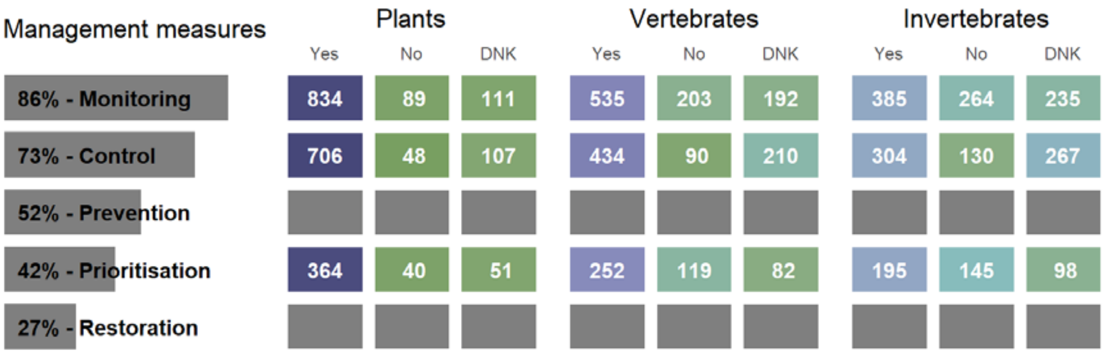

Non-EU Member States (n = 640)

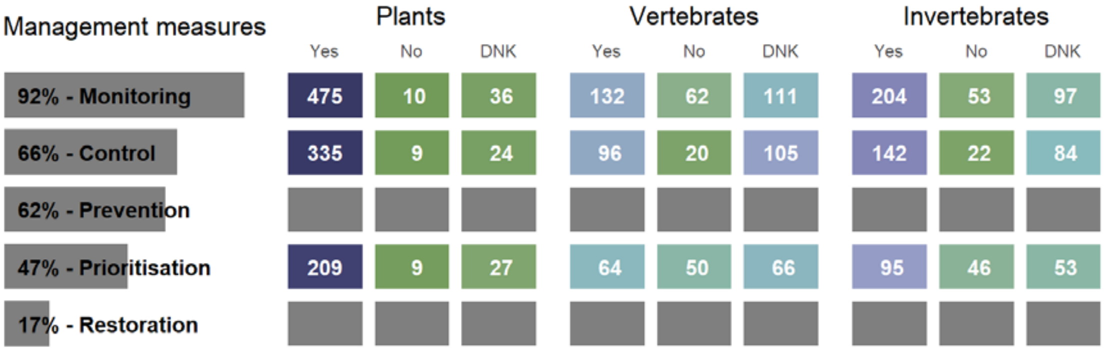

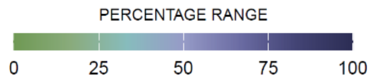

B. Environments

Marine (n = 227)

Management measures	Plants Yes	Plants No	Plants DNK	Vertebrates Yes	Vertebrates No	Vertebrates DNK	Invertebrates Yes	Invertebrates No	Invertebrates DNK
91% - Monitoring	77	23	44	86	24	38	92	23	30
62% - Control	45	20	31	39	17	34	38	19	36
56% - Prevention									
53% - Prioritisation	37	20	24	34	23	29	36	23	25
27% - Restoration									

Coastland (n = 41)

Management measures	Plants Yes	Plants No	Plants DNK	Vertebrates Yes	Vertebrates No	Vertebrates DNK	Invertebrates Yes	Invertebrates No	Invertebrates DNK
83% - Monitoring	26	0	3	10	6	8	1	9	12
78% - Control	27	0	3	12	3	7	4	5	12
41% - Prevention									
39% - Prioritisation	9	1	1	3	2	4	3	2	4
41% - Restoration									

Freshwater (n = 199)

Management measures	Plants Yes	Plants No	Plants DNK	Vertebrates Yes	Vertebrates No	Vertebrates DNK	Invertebrates Yes	Invertebrates No	Invertebrates DNK
89% - Monitoring	98	15	25	97	15	19	77	21	31
67% - Control	72	7	17	68	7	19	35	16	34
48% - Prevention									
40% - Prioritisation	48	6	7	39	15	6	33	14	11
25% - Restoration									

Terrestrial (n = 355)

Management measures	Plants Yes	Plants No	Plants DNK	Vertebrates Yes	Vertebrates No	Vertebrates DNK	Invertebrates Yes	Invertebrates No	Invertebrates DNK
86% - Monitoring	238	13	13	66	42	44	83	44	45
66% - Control	183	3	16	58	7	50	66	13	46
46% - Prevention									
33% - Prioritisation	85	3	7	30	15	16	30	19	18
24% - Restoration									

Urban (n = 97)

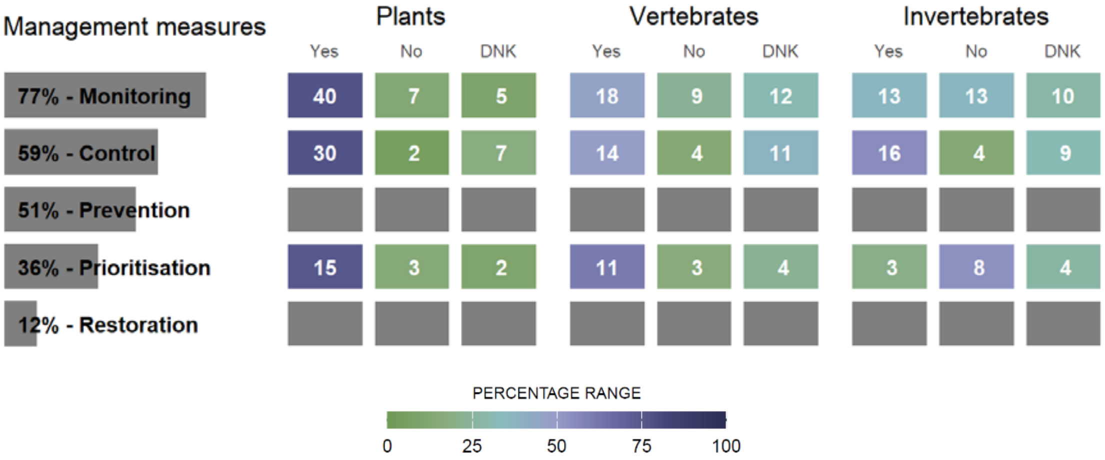
Management measures
Plants
Vertebrates
Invertebrates
Yes
No
DNK
Yes
No
DNK
Yes
No
DNK
77% - Monitoring
40
7
5
18
9
12
13
13
10
59% - Control
30
2
7
14
4
11
16
4
9
51% - Prevention
36% - Prioritisation
15
3
2
11
3
4
3
8
4
12% - Restoration
PERCENTAGE RANGE
0
25
50
75
100

C. Protection of the area

Protected (n = 470)

Management measures	Plants Yes	Plants No	Plants DNK	Vertebrates Yes	Vertebrates No	Vertebrates DNK	Invertebrates Yes	Invertebrates No	Invertebrates DNK
93% - Monitoring	338	34	31	193	81	62	121	113	83
71% - Control	262	12	26	147	30	60	86	50	83
46% - Prevention									
38% - Prioritisation	122	14	14	72	32	26	42	48	38
29% - Restoration									

Partially protected (n = 879)

Management measures	Plants Yes	Plants No	Plants DNK	Vertebrates Yes	Vertebrates No	Vertebrates DNK	Invertebrates Yes	Invertebrates No	Invertebrates DNK
89% - Monitoring	593	32	76	342	98	164	308	123	165
76% - Control	510	28	69	296	54	173	249	79	184
62% - Prevention									
52% - Prioritisation	311	24	41	190	96	82	172	100	83
26% - Restoration									

Unprotected (n = 409)

Management measures	Plants Yes	Plants No	Plants DNK	Vertebrates Yes	Vertebrates No	Vertebrates DNK	Invertebrates Yes	Invertebrates No	Invertebrates DNK
88% - Monitoring	271	20	28	86	55	53	122	54	52
68% - Control	221	15	24	64	21	66	95	18	62
61% - Prevention									
40% - Prioritisation	107	7	19	31	28	33	52	29	23
16% - Restoration									

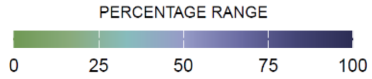

Figure S1.6
Percentage of survey respondents monitoring: (A) the number of invasive alien species (abbreviated IAS), (B) the area occupied by IAS, and (C) the impacts generated by IAS across taxonomic groups. Data derived from questions Q11 to Q13 of the survey (Supplementary Material 2), using the continental vs. marine dataset for panels A and B, and the original dataset for panel C.

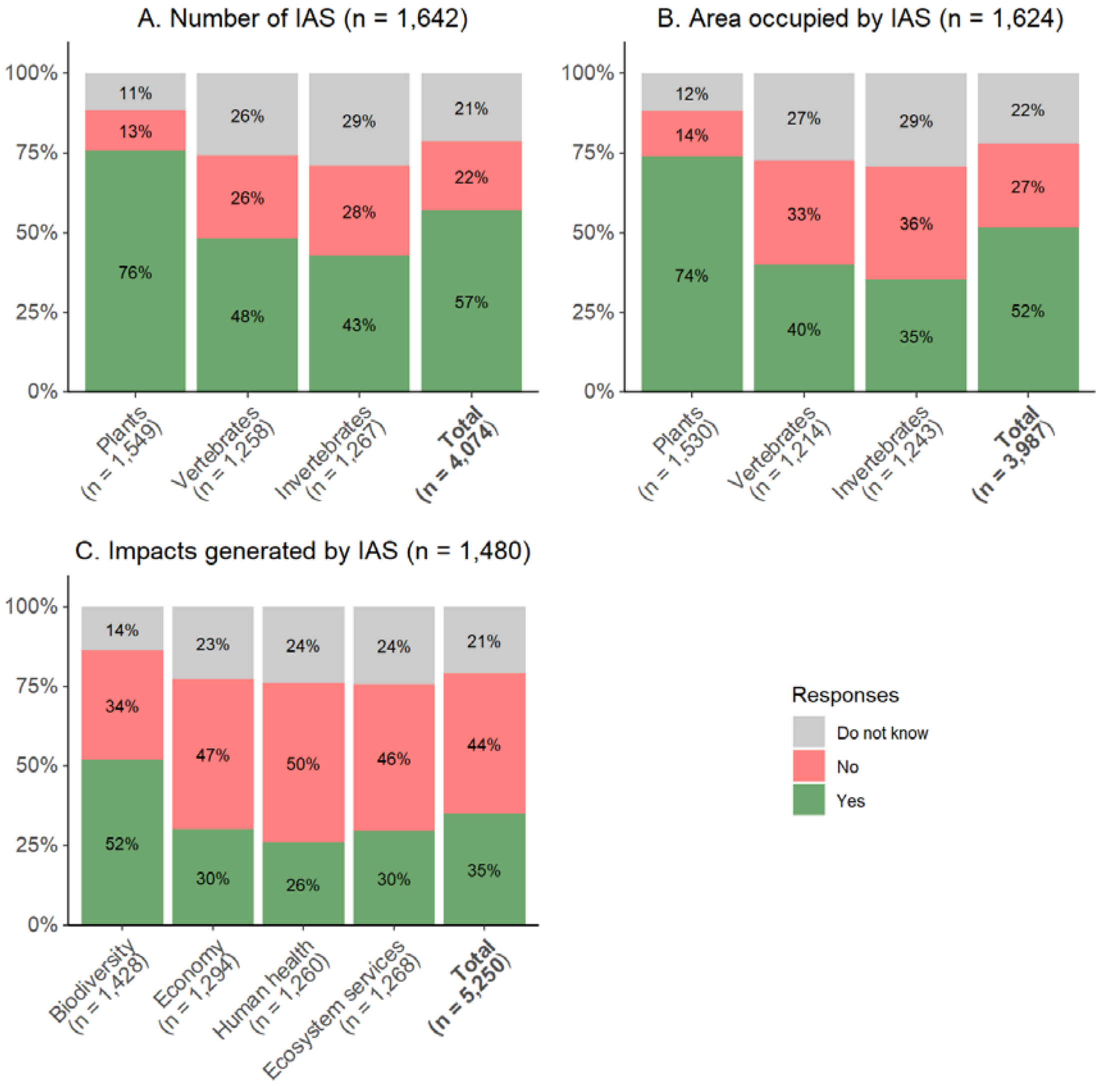

Figure S1.7

Proportion of control and eradication practices on invasive alien species (abbreviated IAS) by taxonomic groups based on survey respondents. Charts show: (A) trends in control practices; (B) attempts to eradicate populations; (C) success of eradication practices; and (D) attempts to eradicate populations before their establishment (i.e. rapid eradication). In panel C, respondents were allowed to select multiple responses, which explains why the number of responses on the x-axis may exceed the number of participants indicated in the title. Data derived from questions Q17, Q18 and Q19 of the survey (Supplementary Material 2), using the continental vs. marine dataset.

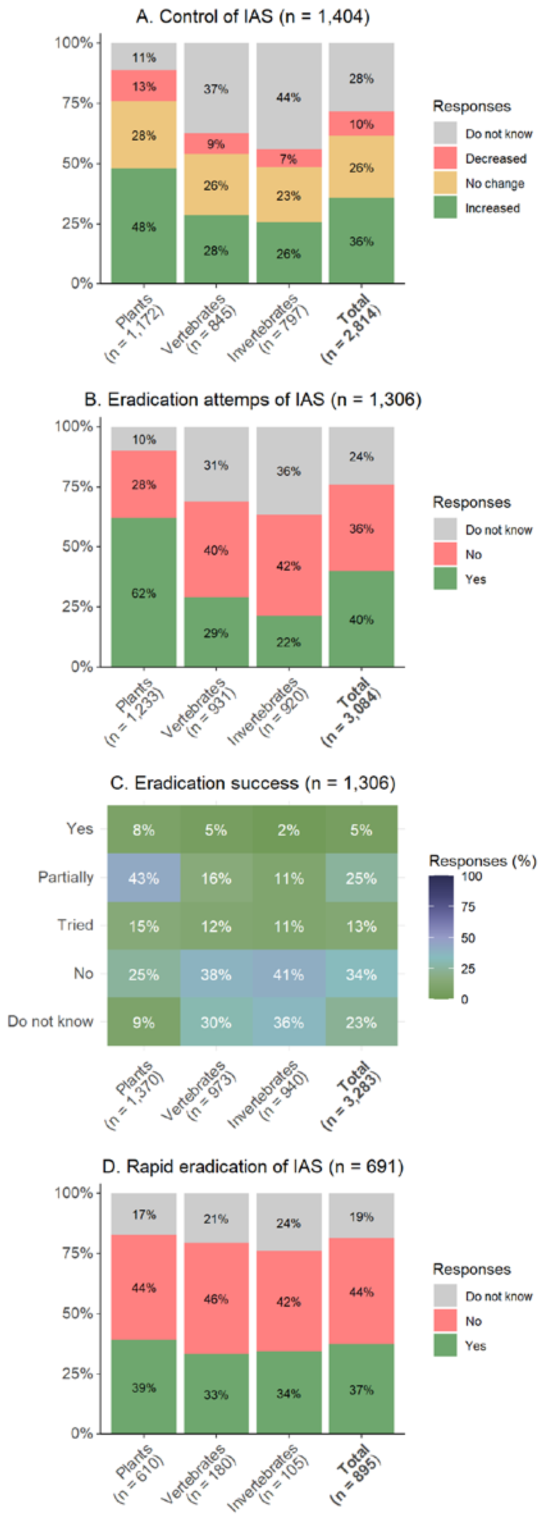

Figure S1.8
Percentage of survey respondents that have (A) a priority list of invasive alien species (abbreviated IAS), and (B) priorities for managing invaded sites across taxonomic groups. Data derived from questions Q14 and Q15 of the survey (Supplementary Material 2), using the continental vs. marine dataset.

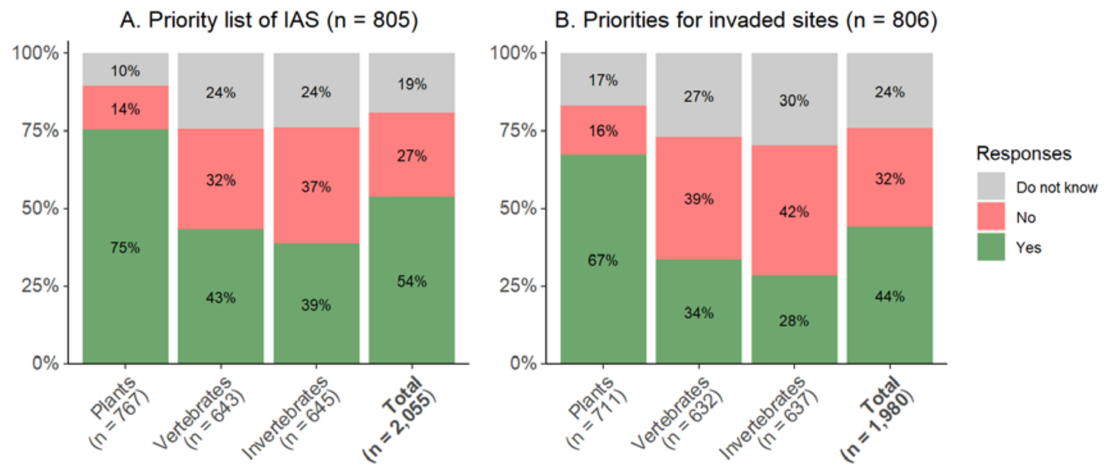

Figure S1.9
Multiple correspondence analysis (MCA) examining the relationship between management measures and the environment (A and B) or the protection of the area (C) across taxonomic groups. Two different sets of environments are considered: (A) continental vs. marine, and (B) further differentiating continental into urban, terrestrial, freshwater, and coastland. Protection of the area differentiates between protected, partially protected, and non-protected areas. Main variables in the plots are depicted in **dark purple**, while supplementary variables are represented in **soft green**. Data derived from questions Q4 (for environment), Q9 (management measures), and Q23 (for protection of the area), except for plots assessing taxonomic groups where questions Q11 and Q12 (monitoring), Q14 and Q15 (prioritisation), and Q17 (control) of the survey (Supplementary Material 2) were used instead of Q9. The continental vs. marine dataset was used in plots A and C, while the specific dataset in plots B. The abbreviations refer to monitoring (mon), prevention (pre), prioritisation (pri), control (con), and restoration (res).

A. Continental and marine

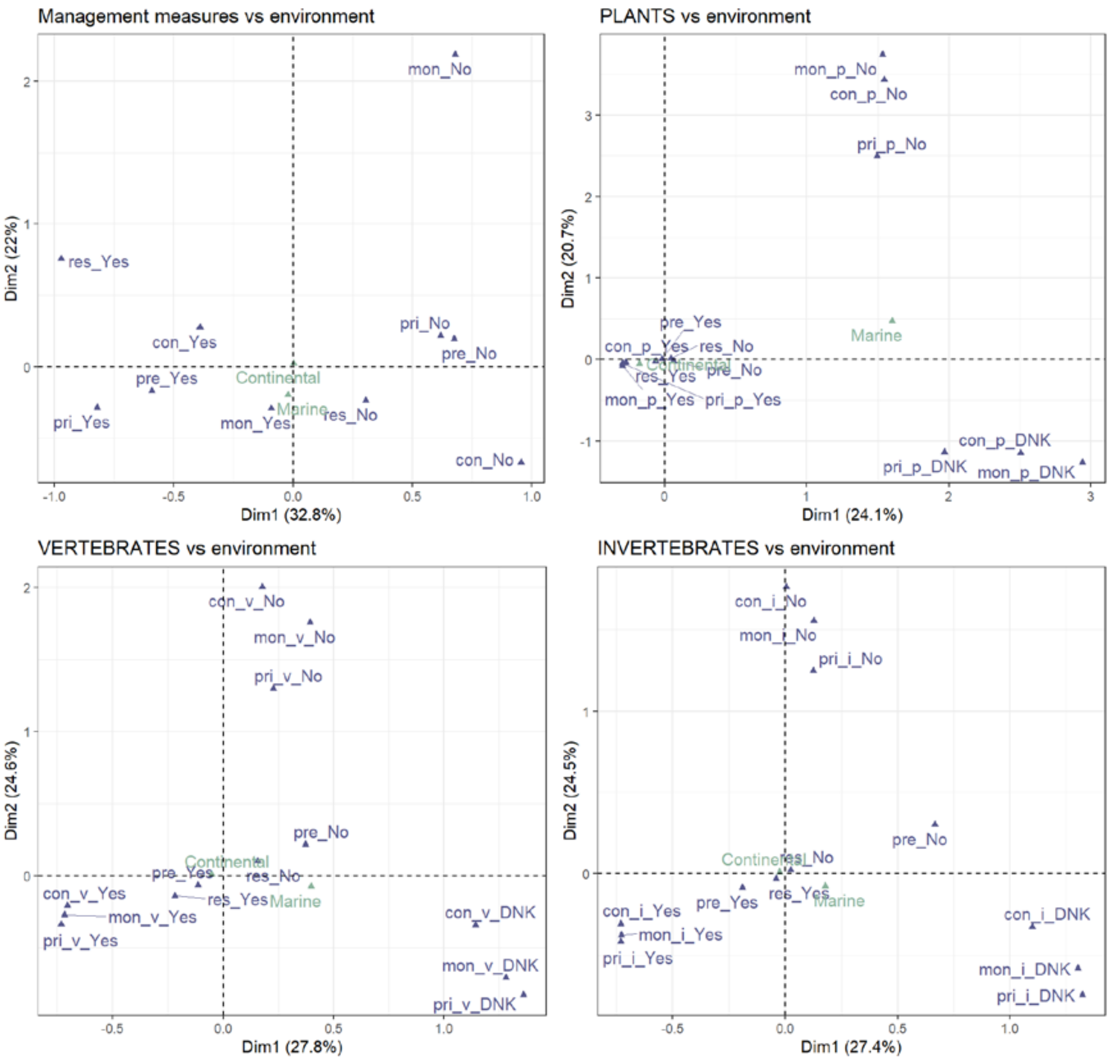

B. Urban, terrestrial, freshwater, coastland, and marine

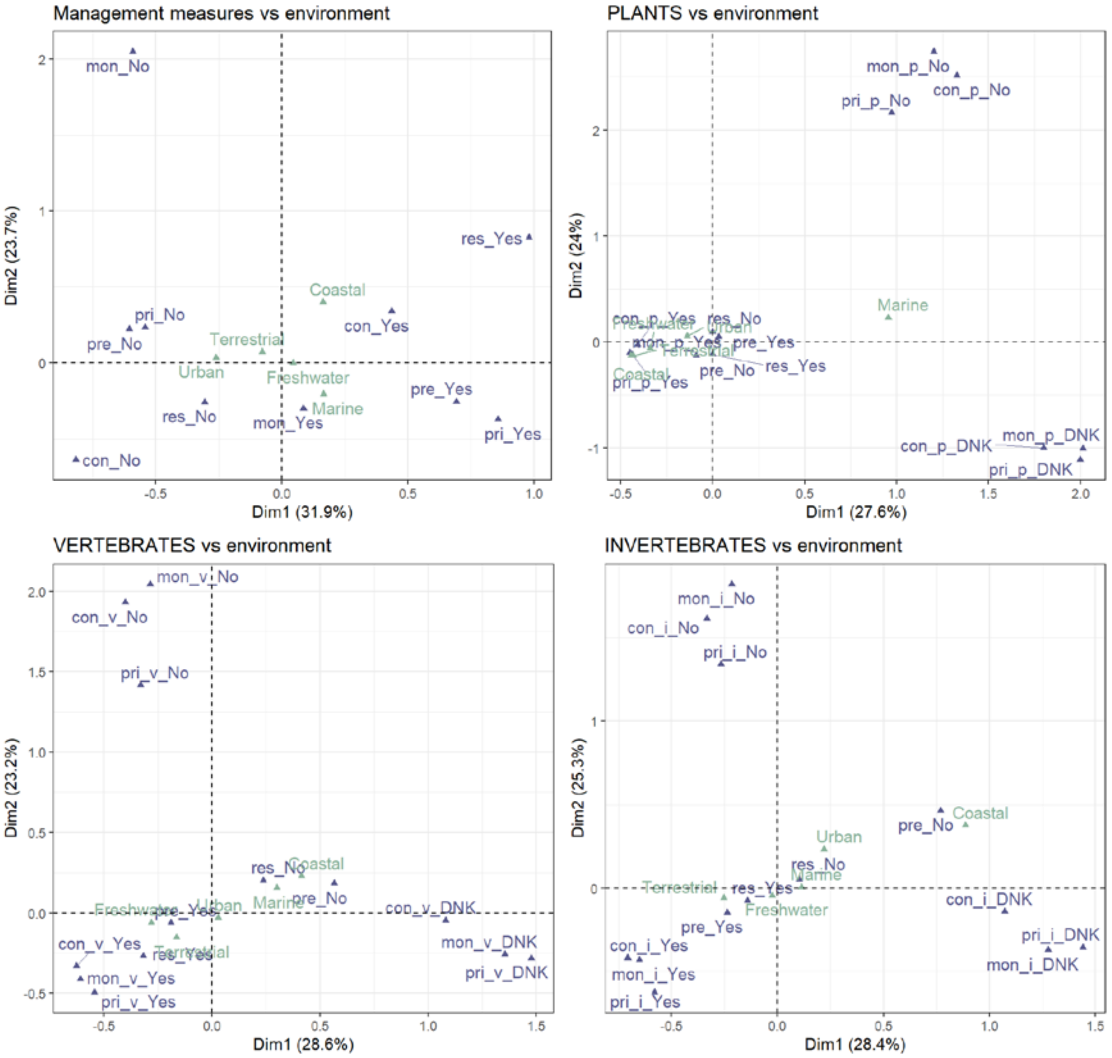

C. Protected, partially protected, and non-protected areas

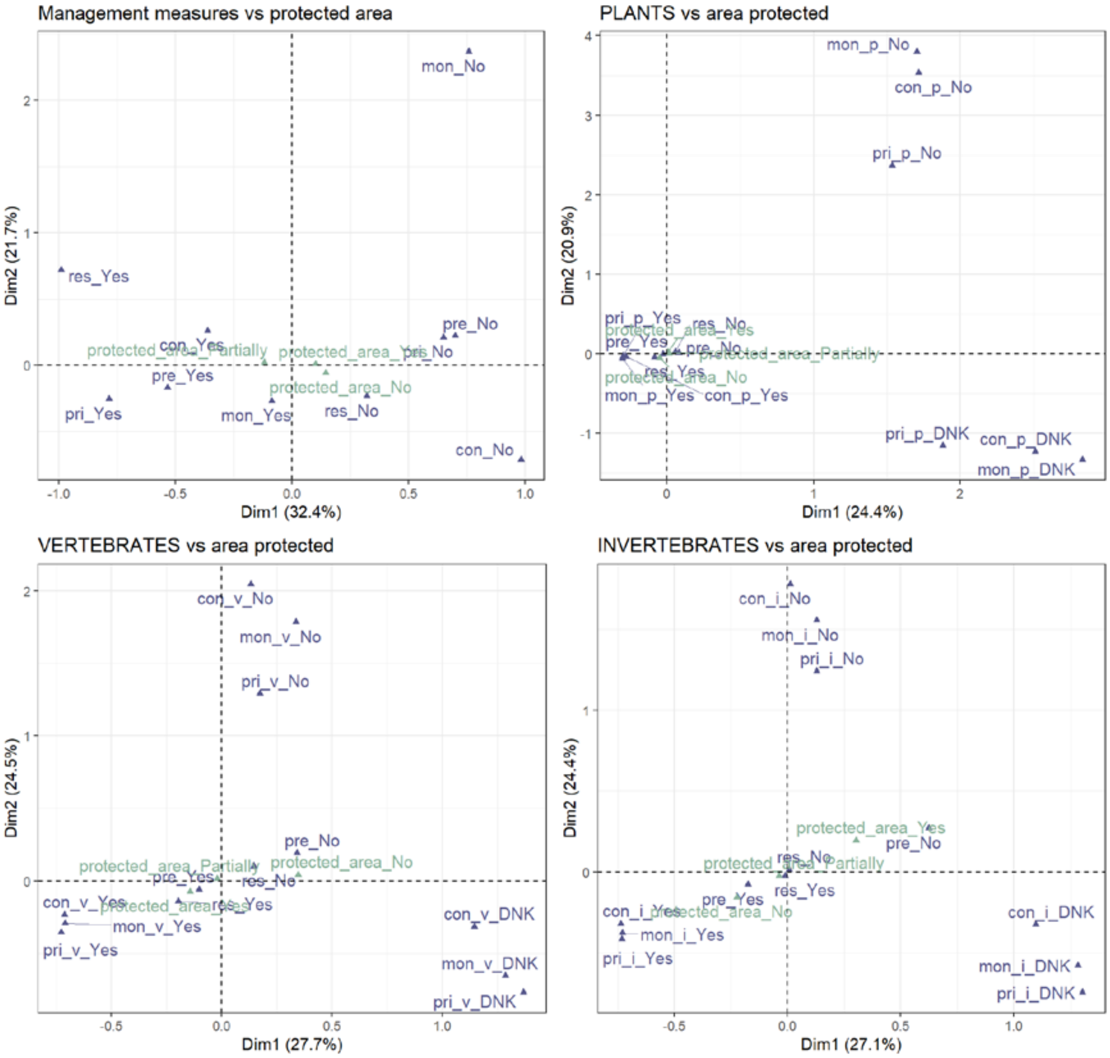

Figure S1.10
Trends in invasive alien species (abbreviated IAS) across taxonomic groups in EU and non-EU Member States. Charts show: (A) trends in the number of IAS; (B) trends in the area occupied by IAS; (C) proportion of impacts from IAS on different sectors; and (D) trends in the impact of IAS on biodiversity. The number of respondents for each question is indicated in the graph titles, while the “n” in the x-axis represents responses for each taxonomic group. Data derived from questions Q5 to Q8 of the survey (Supplementary Material 2), using the continental vs. marine dataset in plots A, B and D, and the original dataset in plots C.

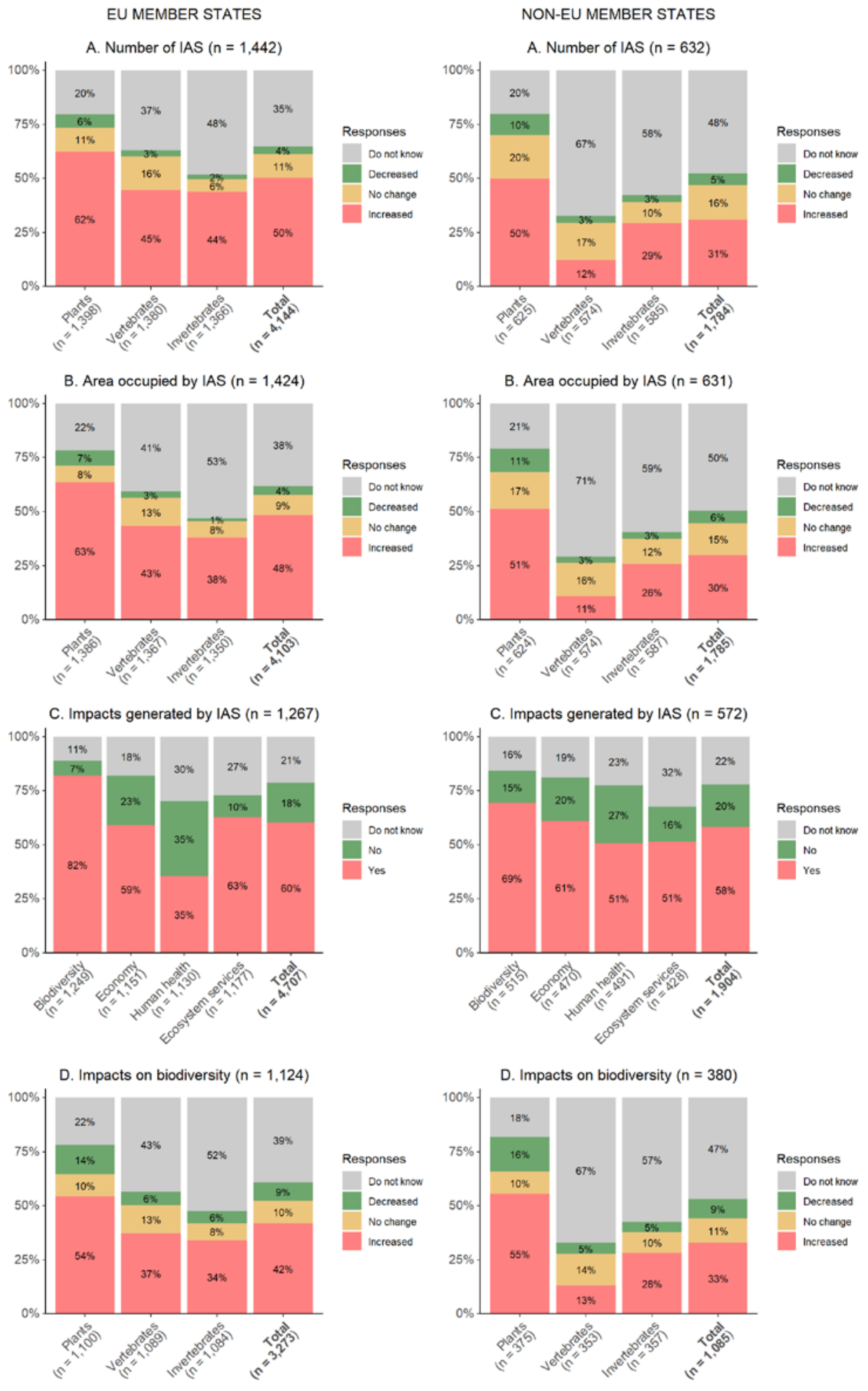

Figure S1.11

Heatmaps showing the relationship between trends in the number of invasive alien species (abbreviated IAS) and the area they occupy by taxonomic groups. The tables on the left panel use the number of species as a benchmark, while the panel on the right focus on the area occupied by IAS. This dual-panel approach facilitates the analysis of how selecting a category based on one variable (number of species or area occupied) influences the distribution observed in the other. Data derived from questions Q5 and Q6 of the survey (Supplementary Material 2), using the continental vs. marine dataset. Chi-squared and p-values computed based on 2,000 Monte-Carlo simulations.

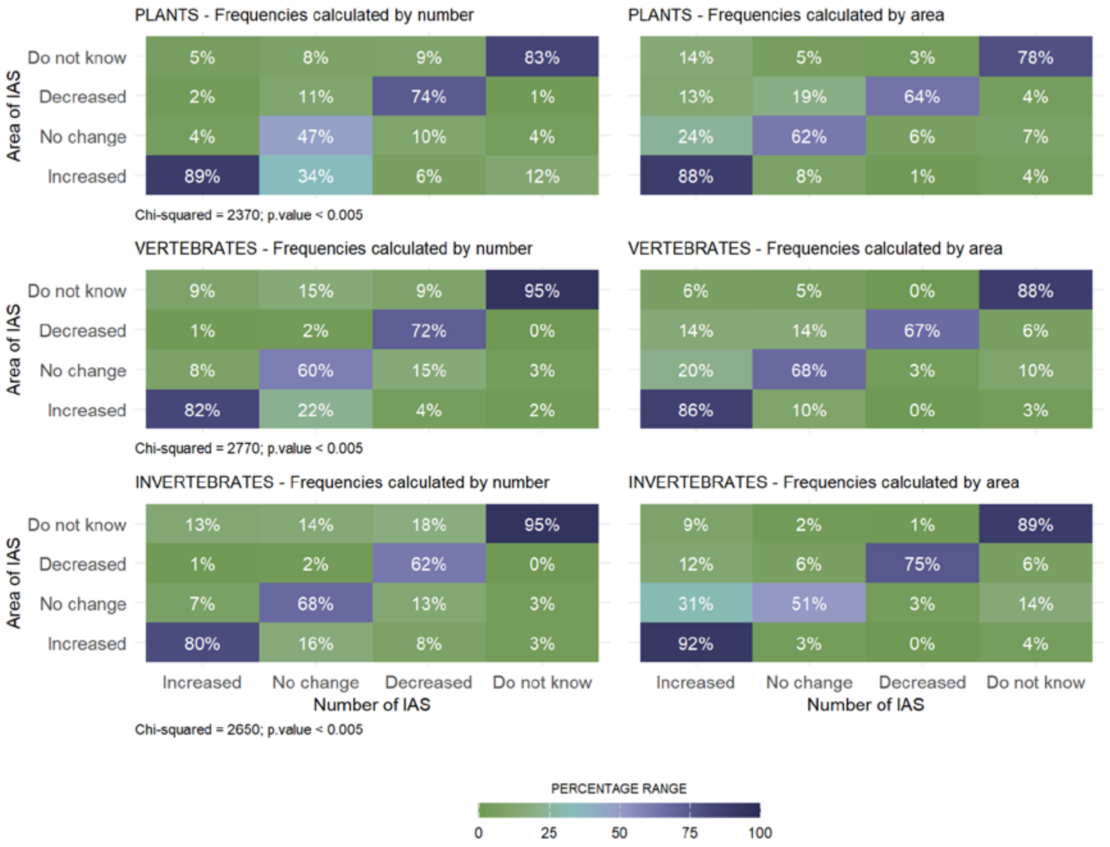

Figure S1.12
Heatmaps showing the relationship between trends in the number of invasive alien species (left panel) or their impact on biodiversity (right panel) and the environment by taxonomic groups. Note that the number of invasive alien species and the area they occupy are highly correlated (Figure S1.11), so the results for the number of invasive alien species are representative of both variables. Data derived from questions Q4, Q5, and Q8 of the survey (Supplementary Material 2), using the continental vs. marine dataset. Chi-squared and p-values computed based on 2,000 Monte-Carlo simulations.

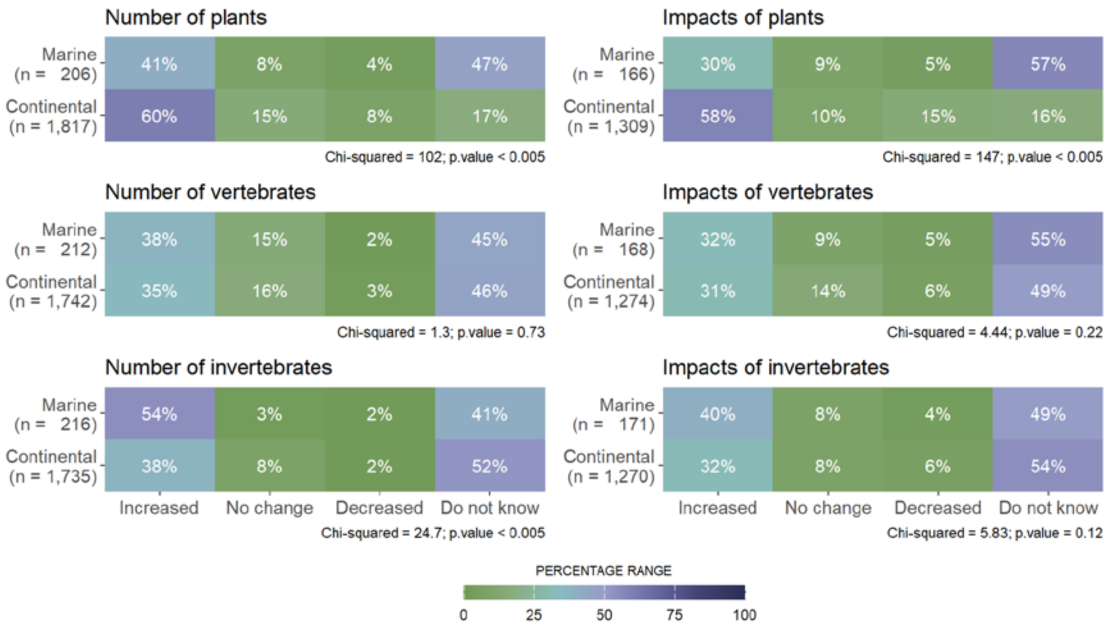

Figure S1.13

Heatmaps showing the relationship between trends in the number of invasive alien species (left panel) or their impact on biodiversity (right panel) and the protection of the area across taxonomic groups. Note that the number of invasive alien species and the area they occupy are highly correlated (Figure S1.11), so the results for the number are representative of both variables. Data derived from questions Q5, Q8 and Q23 of the survey (Supplementary Material 2), using the continental vs. marine dataset. Chi-squared and p-values computed based on 2,000 Monte-Carlo simulations.

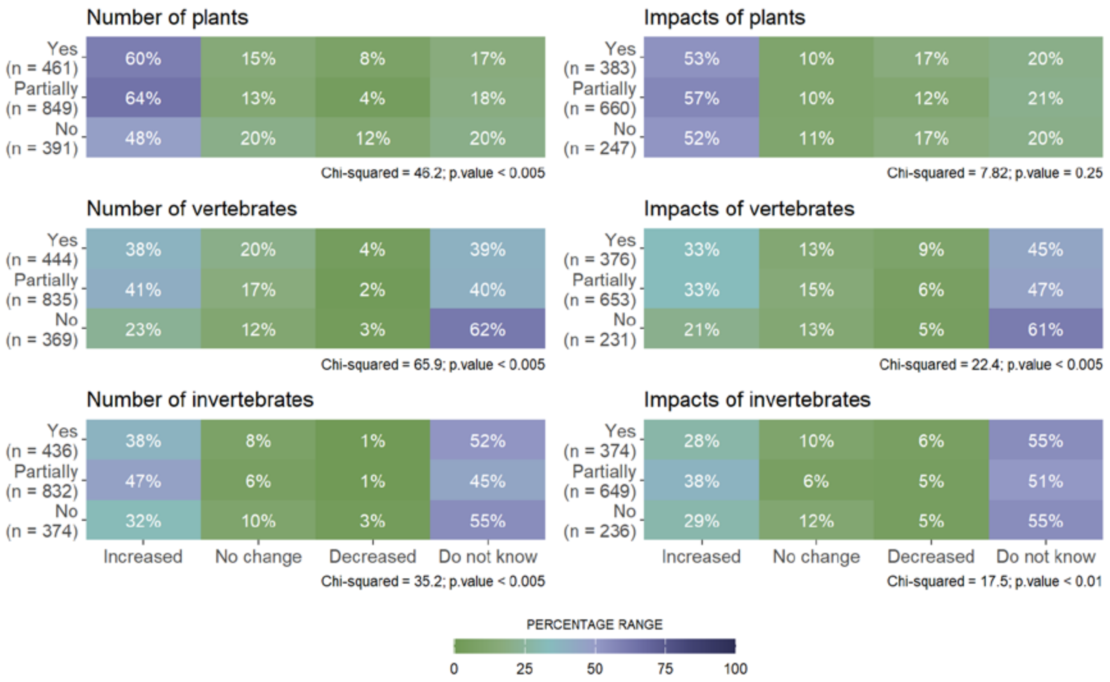

Figure S1.14

Parameters of the multiple correspondence analyses (MCA) examining the relationship between the management measures and the trends in invasive alien species across taxonomic groups, considering both the number of species (left panel) and their impact on biodiversity (right panel) (see Figure 6). Each table shows the contribution of each dimension in the first three rows, followed by the weight of each variable for each dimension. Data derived from questions Q5 (for number of invasive alien species), Q8 (impacts on biodiversity), Q9 (prevention and restoration), Q11 and Q12 (monitoring), Q14 and Q15 (prioritisation), and Q17 (control) of the survey (Supplementary Material 2), using the continental vs. marine dataset. The abbreviations refer to monitoring (mon), prevention (pre), prioritisation (pri), control (con), and restoration (res).

Number in PLANTS

	Dim1	Dim2	Dim3	Dim4	Dim5
eigenvalue	0.33	0.23	0.17	0.16	0.11
% of variance	32.56	23.48	17.18	15.70	11.08
cumulative % of variance	32.56	56.04	73.22	88.92	100.00
	Dim1	Dim2	Dim3	Dim4	Dim5
res_No	0.11	0.62	-0.36	-0.38	-0.03
res_Yes	-0.16	-0.92	0.53	0.57	0.05
pre_No	0.23	1.40	0.81	0.86	0.02
pre_Yes	-0.07	-0.41	-0.24	-0.25	-0.01
mon_No	3.87	-0.58	-0.18	0.39	-2.68
mon_Yes	-0.17	0.03	0.01	-0.02	0.12
pri_No	2.44	-0.26	2.04	-1.80	0.95
pri_Yes	-0.17	0.02	-0.14	0.13	-0.07
con_No	4.05	-0.40	-2.44	1.90	2.33
con_Yes	-0.13	0.01	0.08	-0.06	-0.07

Impacts in PLANTS

	Dim1	Dim2	Dim3	Dim4	Dim5
eigenvalue	0.33	0.24	0.17	0.15	0.11
% of variance	32.81	23.84	16.86	15.31	11.18
cumulative % of variance	32.81	56.66	73.52	88.82	100.00
	Dim1	Dim2	Dim3	Dim4	Dim5
res_No	0.12	0.67	-0.37	-0.41	-0.03
res_Yes	-0.15	-0.87	0.48	0.53	0.04
pre_No	0.20	1.39	0.84	0.80	-0.02
pre_Yes	-0.06	-0.42	-0.25	-0.24	0.01
mon_No	3.69	-0.60	-0.01	0.13	-2.61
mon_Yes	-0.18	0.03	0.00	-0.01	0.13
pri_No	2.46	-0.33	1.77	-1.56	1.16
pri_Yes	-0.19	0.03	-0.14	0.12	-0.09
con_No	3.71	-0.16	-2.45	2.15	1.89
con_Yes	-0.13	0.01	0.09	-0.08	-0.07

Number in VERTEBRATES

	Dim1	Dim2	Dim3	Dim4	Dim5
eigenvalue	0.42	0.23	0.16	0.12	0.07
% of variance	41.53	23.27	16.43	11.81	69.49
cumulative % of variance	41.53	64.81	81.24	93.05	100.00
	Dim1	Dim2	Dim3	Dim4	Dim5
res_No	0.15	0.66	-0.50	0.15	-0.04
res_Yes	-0.20	-0.90	0.68	-0.20	0.06
pre_No	0.47	1.41	1.30	-0.19	-0.01
pre_Yes	-0.12	-0.36	-0.33	0.05	0.00
mon_No	1.61	-0.35	0.01	-0.03	-0.85
mon_Yes	-0.47	0.10	0.00	0.01	0.25
pri_No	1.12	-0.03	-0.28	-0.73	0.37
pri_Yes	-0.56	0.01	0.14	0.36	-0.19
con_No	1.69	-0.33	0.25	1.15	0.55
con_Yes	-0.36	0.07	-0.05	-0.25	-0.12

Impacts in VERTEBRATES

	Dim1	Dim2	Dim3	Dim4	Dim5
eigenvalue	0.40	0.24	0.17	0.13	0.07
% of variance	39.50	23.63	16.69	12.86	73.14
cumulative % of variance	39.50	63.13	79.82	92.69	100.00
	Dim1	Dim2	Dim3	Dim4	Dim5
res_No	0.08	0.71	-0.50	0.21	-0.06
res_Yes	-0.10	-0.88	0.62	-0.26	0.07
pre_No	0.38	1.41	1.29	-0.30	-0.02
pre_Yes	-0.10	-0.36	-0.33	0.08	0.00
mon_No	1.63	-0.30	-0.03	-0.12	-0.87
mon_Yes	-0.46	0.09	0.01	0.03	0.25
pri_No	1.15	0.05	-0.38	-0.69	0.45
pri_Yes	-0.53	-0.02	0.17	0.32	-0.21
con_No	1.70	-0.29	0.42	1.31	0.51
con_Yes	-0.33	0.06	-0.08	-0.26	-0.10

Number in INVERTEBRATES

	Dim1	Dim2	Dim3	Dim4	Dim5
eigenvalue	0.43	0.22	0.17	0.11	0.08
% of variance	42.66	21.67	16.94	10.68	8.05
cumulative % of variance	42.66	64.33	81.27	91.95	100.00
	Dim1	Dim2	Dim3	Dim4	Dim5
res_No	-0.01	0.67	-0.43	0.04	0.00
res_Yes	0.01	-1.05	0.68	-0.06	0.00
pre_No	0.85	1.25	1.54	0.18	0.18
pre_Yes	-0.18	-0.26	-0.33	-0.04	-0.04
mon_No	1.23	-0.21	-0.28	-0.16	0.72
mon_Yes	-0.56	0.10	0.13	0.08	-0.33
pri_No	1.08	0.04	-0.04	-0.60	-0.45
pri_Yes	-0.62	-0.02	0.02	0.35	0.26
con_No	1.37	-0.28	-0.22	0.97	-0.35
con_Yes	-0.45	0.09	0.07	-0.32	0.11

Impacts in INVERTEBRATES

	Dim1	Dim2	Dim3	Dim4	Dim5
eigenvalue	0.41	0.22	0.17	0.11	0.09
% of variance	40.92	21.58	17.39	11.38	87.19
cumulative % of variance	40.92	62.50	79.90	91.28	100.00
	Dim1	Dim2	Dim3	Dim4	Dim5
res_No	-0.01	0.71	-0.46	0.09	0.00
res_Yes	0.02	-0.98	0.63	-0.12	0.01
pre_No	0.84	1.21	1.57	0.22	0.22
pre_Yes	-0.18	-0.26	-0.33	-0.05	-0.05
mon_No	1.16	-0.18	-0.31	-0.22	0.70
mon_Yes	-0.58	0.09	0.15	0.11	-0.35
pri_No	0.99	0.10	-0.04	-0.53	-0.47
pri_Yes	-0.66	-0.06	0.02	0.35	0.32
con_No	1.28	-0.37	-0.19	0.98	-0.29
con_Yes	-0.45	0.13	0.07	-0.34	0.10

PANELS

Panel S1.1

Description of the process used to separate responses according to the environment in which respondents conduct their management practices on invasive alien species (abbreviated IAS), and analysis of responses according to the characteristic of the questions under study.

Respondents were asked to provide information about their work environment by answering question Q4 of the survey (Supplementary Material 2). They had to select from 10 environments, with the option of selecting multiple environments.

Q4

Select the environments that characterize your management area most accurately.

Select as many options as necessary.

Continental

[ ] Urban areas

[ ] Forestry (forests, shrubland, sparsely vegetated land, etc.)

[ ] Grasslands

[ ] Croplands

[ ] Rivers

[ ] Lakes and inner wetlands

[ ] Hydraulic structure (channels, dams, etc.)

[ ] Terrestrial coastal areas (cliffs, salt marshes, lagoons, dry beaches, dunes, etc.)

Marine

[ ] Marine coastal areas (intertidal zone, estuaries, shelf, etc.)

[ ] Oceans

Certain questions of the survey, primarily designed to assess differences between taxonomic groups (Q5-Q8, Q11-Q15, Q17-Q19, Supplementary Material 2), could be answered separately for two broad environmental categories. These categories were continental (including urban areas, forests, grasslands, croplands, rivers, lakes and inner wetlands, hydraulic structure, or terrestrial coastal areas) and marine environments (comprising marine coastal areas and oceans exclusively). To accomplish this, we designed a survey with conditional or cascading questions. Depending on the environments selected in Q4, specific tables requesting information in one or two environment categories would appear in subsequent questions. For example, in the case of Q5 "How has the number of IAS (in different taxonomic groups) changed?", if respondents selected a continental environment, they would always respond to Table type A. Similarly, those who selected marine environments would consistently respond to Table type B. Lastly, participants who selected both environments would respond to questions based on Table type C, which presents taxonomic groups categorised by environment.

Table type A appears when respondents selected CONTINENTAL environments in Q4:

	Decreased	No change	Increased	Do not know	Not applicable
Plants	[ ]	[ ]	[ ]	[ ]	[ ]
Invertebrates	[ ]	[ ]	[ ]	[ ]	[ ]
Vertebrates	[ ]	[ ]	[ ]	[ ]	[ ]
Other (specify, e.g. "fungi"):	[ ]	[ ]	[ ]	[ ]	[ ]

Table type B appears when respondents selected **MARINE** environments in Q4:

	Decreased	No change	Increased	Do not know	Not applicable
Plants	[ ]	[ ]	[ ]	[ ]	[ ]
Invertebrates	[ ]	[ ]	[ ]	[ ]	[ ]
Vertebrates	[ ]	[ ]	[ ]	[ ]	[ ]
Other (specify, e.g. "fungi"):	[ ]	[ ]	[ ]	[ ]	[ ]

Table type C appears when respondents selected both, **CONTINENTAL and MARINE**, environments in Q4:

	Decreased	No change	Increased	Do not know	Not applicable
Continental Plants	[ ]	[ ]	[ ]	[ ]	[ ]
Continental Invertebrates	[ ]	[ ]	[ ]	[ ]	[ ]
Continental Vertebrates	[ ]	[ ]	[ ]	[ ]	[ ]
Continental Other (specify, e.g. "fungi"):	[ ]	[ ]	[ ]	[ ]	[ ]
Marine Plants	[ ]	[ ]	[ ]	[ ]	[ ]
Marine Invertebrates	[ ]	[ ]	[ ]	[ ]	[ ]
Marine Vertebrates	[ ]	[ ]	[ ]	[ ]	[ ]
Marine Other (specify, e.g. "fungi"):	[ ]	[ ]	[ ]	[ ]	[ ]

Given the dependence of certain questions on the environment and the taxonomic group, the number of responses varied across the survey, as did the number of participants who dropped out or skipped questions. For the following analyses, only those participants who responded up to question Q9 were considered, a total of 1,928 participants, representing 72% of those who started the survey (Figure S1.2). Among these participants, their responses to Q4 were distributed as follows:

Environment reported in Q4	Responses	%
Forest	113	5.9
Cropland	101	5.2
Urban	96	5.0
Urban & Forest & Grassland & Cropland	81	4.2
Urban & Forest & Grassland & Cropland & River & Lakes/wetlands & Hydraulic	79	4.1
Urban & Forest & Grassland & Cropland & River & Lakes/wetlands	69	3.6
River	58	3.0
Urban & Forest	42	2.2
Forest & Grassland	41	2.1
Lakes/wetlands	41	2.1
Marine coast	41	2.1
River & Lakes/wetlands & Hydraulic	40	2.1
Urban & Forest & Grassland & Cropland & River	40	2.1
Urban & Forest & Cropland	39	2.0
Forest & Grassland & Cropland	38	2.0
Terrestrial coast	31	1.6
Urban & Forest & Grassland	31	1.6
Forest & Grassland & River & Lakes/wetlands	30	1.6
River & Lakes/wetlands	29	1.5
Forest & Cropland	28	1.5
Forest & Grassland & Cropland & River	28	1.5
Forest & Grassland & Cropland & River & Lakes/wetlands	24	1.2

Environment reported in Q4	Responses	%
Grassland	23	1.2
Forest & Grassland & Cropland & River & Lakes/wetlands & Hydraulic	21	1.1
Marine coast & Ocean	19	1.0
Forest & Grassland & River	18	0.9
Forest & Terrestrial coast	18	0.9
Forest & Grassland & Lakes/wetlands	17	0.9
Urban & Forest & River	17	0.9
Urban & Forest & Grassland & Cropland & River & Lakes/wetlands & Hydraulic & Marine coast & Terrestrial coast	16	0.8
Urban & Forest & Grassland & Cropland & River & Lakes/wetlands & Hydraulic & Terrestrial coast	16	0.8
Forest & Grassland & Cropland & Lakes/wetlands	15	0.8
Urban & Forest & Grassland & River & Lakes/wetlands	15	0.8
Urban & Grassland & Cropland	15	0.8
Forest & Lakes/wetlands	13	0.7
Urban & Cropland	13	0.7
Urban & Forest & Grassland & Cropland & River & Lakes/wetlands & Hydraulic & Marine coast & Terrestrial coast & Ocean	13	0.7
Forest & Cropland & River	12	0.6
Urban & Forest & Grassland & River	12	0.6
Urban & River & Lakes/wetlands	11	0.6
Forest & River	10	0.5
Grassland & Cropland	10	0.5
Urban & Forest & Grassland & Cropland & Lakes/wetlands	10	0.5
Urban & Forest & Grassland & Lakes/wetlands	10	0.5
Forest & River & Lakes/wetlands	9	0.5
Grassland & River	8	0.4
Urban & Forest & Cropland & River	8	0.4
Urban & Forest & Grassland & Cropland & River & Lakes/wetlands & Marine coast & Terrestrial coast	8	0.4
Urban & River	8	0.4
Urban & River & Lakes/wetlands & Hydraulic	8	0.4
Forest & Grassland & Cropland & River & Hydraulic	7	0.4
Forest & Grassland & River & Lakes/wetlands & Hydraulic	7	0.4
Grassland & Cropland & River & Lakes/wetlands	7	0.4
Grassland & River & Lakes/wetlands	7	0.4
Marine coast & Terrestrial coast	7	0.4
River & Hydraulic	7	0.4
Urban & Forest & Grassland & River & Lakes/wetlands & Hydraulic	7	0.4
Urban & Forest & Grassland & River & Lakes/wetlands & Terrestrial coast	7	0.4
Urban & Grassland & River	7	0.4
Forest & Grassland & River & Lakes/wetlands & Terrestrial coast	6	0.3
Forest & Grassland & Terrestrial coast	6	0.3
Grassland & Lakes/wetlands	6	0.3
Grassland & Lakes/wetlands & Terrestrial coast	6	0.3
Hydraulic	6	0.3
Urban & Forest & Grassland & Cropland & River & Lakes/wetlands & Terrestrial coast	6	0.3
Urban & Grassland & Cropland & River & Lakes/wetlands	6	0.3
Forest & Grassland & Lakes/wetlands & Marine coast & Terrestrial coast	5	0.3
Forest & Marine coast & Terrestrial coast	5	0.3
Lakes/wetlands & Hydraulic	5	0.3
River & Lakes/wetlands & Terrestrial coast	5	0.3
Urban & Forest & Grassland & Cropland & River & Hydraulic	5	0.3
Urban & Forest & Grassland & Cropland & Terrestrial coast	5	0.3
Cropland & River	4	0.2
Forest & Grassland & Cropland & River & Lakes/wetlands & Hydraulic & Terrestrial coast	4	0.2
Forest & Grassland & Cropland & River & Lakes/wetlands & Marine coast & Terrestrial coast	4	0.2

Environment reported in Q4	Responses	%
Forest & Grassland & Cropland & River & Lakes/wetlands & Terrestrial coast	4	0.2
Forest & Grassland & Lakes/wetlands & Terrestrial coast	4	0.2
Forest & Grassland & River & Hydraulic	4	0.2
Forest & Grassland & River & Lakes/wetlands & Marine coast & Terrestrial coast	4	0.2
Forest & Lakes/wetlands & Marine coast & Terrestrial coast	4	0.2
Grassland & Cropland & Lakes/wetlands	4	0.2
Ocean	4	0.2
Urban & Forest & Cropland & River & Lakes/wetlands	4	0.2
Urban & Forest & Grassland & Cropland & Hydraulic	4	0.2
Urban & Forest & Grassland & Cropland & Lakes/wetlands & Hydraulic	4	0.2
Urban & Forest & Lakes/wetlands	4	0.2
Urban & Forest & Marine coast & Terrestrial coast	4	0.2
Forest & Grassland & Cropland & Lakes/wetlands & Hydraulic	3	0.2
Forest & Grassland & Cropland & Terrestrial coast	3	0.2
Forest & Grassland & Marine coast & Terrestrial coast	3	0.2
Forest & Grassland & River & Lakes/wetlands & Hydraulic & Terrestrial coast	3	0.2
Forest & Grassland & River & Terrestrial coast	3	0.2
Forest & Marine coast & Terrestrial coast & Ocean	3	0.2
Forest & River & Lakes/wetlands & Terrestrial coast	3	0.2
Forest & River & Terrestrial coast	3	0.2
Lakes/wetlands & Terrestrial coast	3	0.2
Marine coast & Terrestrial coast & Ocean	3	0.2
River & Lakes/wetlands & Marine coast & Terrestrial coast	3	0.2
Urban & Cropland & Hydraulic	3	0.2
Urban & Forest & Cropland & River & Hydraulic	3	0.2
Urban & Forest & Grassland & Cropland & Lakes/wetlands & Marine coast & Terrestrial coast	3	0.2
Urban & Forest & Grassland & Cropland & Marine coast & Terrestrial coast	3	0.2
Urban & Forest & Grassland & River & Terrestrial coast	3	0.2
Urban & Forest & River & Lakes/wetlands	3	0.2
Urban & Forest & Terrestrial coast	3	0.2
Urban & Grassland & Lakes/wetlands	3	0.2
Urban & Lakes/wetlands	3	0.2
Urban & River & Hydraulic	3	0.2
Urban & River & Terrestrial coast	3	0.2
Cropland & Hydraulic	2	0.1
Cropland & River & Hydraulic	2	0.1
Cropland & River & Lakes/wetlands & Hydraulic	2	0.1
Forest & Cropland & River & Lakes/wetlands	2	0.1
Forest & Cropland & River & Lakes/wetlands & Hydraulic	2	0.1
Forest & Cropland & River & Lakes/wetlands & Marine coast	2	0.1
Forest & Cropland & River & Lakes/wetlands & Marine coast & Terrestrial coast	2	0.1
Forest & Grassland & Cropland & Lakes/wetlands & Terrestrial coast	2	0.1
Forest & Hydraulic	2	0.1
Forest & Lakes/wetlands & Terrestrial coast	2	0.1
Forest & River & Hydraulic	2	0.1
Forest & River & Lakes/wetlands & Hydraulic	2	0.1
Grassland & Cropland & River	2	0.1
Grassland & Hydraulic	2	0.1
Grassland & Lakes/wetlands & Hydraulic	2	0.1
Grassland & River & Hydraulic	2	0.1
Grassland & Terrestrial coast	2	0.1
River & Lakes/wetlands & Hydraulic & Marine coast	2	0.1
River & Lakes/wetlands & Hydraulic & Marine coast & Ocean	2	0.1
River & Lakes/wetlands & Hydraulic & Marine coast & Terrestrial coast	2	0.1
River & Lakes/wetlands & Marine coast	2	0.1
River & Marine coast	2	0.1
River & Marine coast & Terrestrial coast	2	0.1

Environment reported in Q4	Responses	%
Urban & Cropland & River	2	0.1
Urban & Cropland & River & Lakes/wetlands & Hydraulic	2	0.1
Urban & Forest & Cropland & Lakes/wetlands	2	0.1
Urban & Forest & Cropland & Lakes/wetlands & Hydraulic	2	0.1
Urban & Forest & Cropland & Marine coast	2	0.1
Urban & Forest & Cropland & Terrestrial coast	2	0.1
Urban & Forest & Grassland & River & Marine coast & Terrestrial coast	2	0.1
Urban & Forest & Lakes/wetlands & Marine coast & Terrestrial coast	2	0.1
Urban & Grassland	2	0.1
Urban & Grassland & Cropland & Lakes/wetlands	2	0.1
Urban & Grassland & Cropland & River & Lakes/wetlands & Hydraulic	2	0.1
Urban & Grassland & Cropland & Terrestrial coast	2	0.1
Urban & Hydraulic	2	0.1
Urban & Lakes/wetlands & Terrestrial coast	2	0.1
Cropland & Lakes/wetlands & Hydraulic	1	0.1
Cropland & Lakes/wetlands & Hydraulic & Marine coast & Terrestrial coast	1	0.1
Cropland & River & Lakes/wetlands & Marine coast & Terrestrial coast	1	0.1
Cropland & Terrestrial coast	1	0.1
Forest & Cropland & Hydraulic	1	0.1
Forest & Cropland & Lakes/wetlands & Terrestrial coast	1	0.1
Forest & Cropland & River & Hydraulic	1	0.1
Forest & Cropland & River & Lakes/wetlands & Terrestrial coast	1	0.1
Forest & Cropland & Terrestrial coast	1	0.1
Forest & Grassland & Cropland & Hydraulic	1	0.1
Forest & Grassland & Cropland & Hydraulic & Terrestrial coast	1	0.1
Forest & Grassland & Cropland & Lakes/wetlands & Marine coast & Terrestrial coast	1	0.1
Forest & Grassland & Cropland & River & Lakes/wetlands & Hydraulic & Marine coast & Terrestrial coast	1	0.1
Forest & Grassland & Cropland & River & Lakes/wetlands & Hydraulic & Marine coast & Terrestrial coast & Ocean	1	0.1
Forest & Grassland & Cropland & River & Lakes/wetlands & Marine coast & Terrestrial coast & Ocean	1	0.1
Forest & Grassland & Lakes/wetlands & Hydraulic	1	0.1
Forest & Grassland & River & Hydraulic & Marine coast & Terrestrial coast	1	0.1
Forest & Grassland & River & Hydraulic & Terrestrial coast	1	0.1
Forest & Grassland & River & Lakes/wetlands & Hydraulic & Marine coast & Terrestrial coast & Ocean	1	0.1
Forest & Grassland & River & Lakes/wetlands & Marine coast & Terrestrial coast & Ocean	1	0.1
Forest & Lakes/wetlands & Hydraulic & Terrestrial coast	1	0.1
Forest & Marine coast	1	0.1
Forest & River & Lakes/wetlands & Hydraulic & Marine coast	1	0.1
Forest & River & Lakes/wetlands & Marine coast	1	0.1
Forest & River & Lakes/wetlands & Marine coast & Terrestrial coast & Ocean	1	0.1
Grassland & Cropland & Lakes/wetlands & Marine coast & Terrestrial coast	1	0.1
Grassland & Cropland & River & Hydraulic	1	0.1
Grassland & Cropland & River & Lakes/wetlands & Hydraulic	1	0.1
Grassland & Lakes/wetlands & Marine coast & Terrestrial coast	1	0.1
Grassland & Marine coast & Terrestrial coast	1	0.1
Grassland & River & Lakes/wetlands & Terrestrial coast	1	0.1
Lakes/wetlands & Hydraulic & Marine coast & Terrestrial coast	1	0.1
Lakes/wetlands & Marine coast	1	0.1
Lakes/wetlands & Marine coast & Terrestrial coast	1	0.1
Lakes/wetlands & Marine coast & Terrestrial coast & Ocean	1	0.1
River & Hydraulic & Marine coast	1	0.1
River & Lakes/wetlands & Hydraulic & Marine coast & Terrestrial coast & Ocean	1	0.1
River & Lakes/wetlands & Marine coast & Terrestrial coast & Ocean	1	0.1
River & Lakes/wetlands & Ocean	1	0.1
River & Marine coast & Ocean	1	0.1

**Environment reported in Q4**	**Responses**	**%**
River & Ocean	1	0.1
River & Terrestrial coast	1	0.1
Urban & Cropland & Lakes/wetlands	1	0.1
Urban & Cropland & Lakes/wetlands & Terrestrial coast	1	0.1
Urban & Cropland & Marine coast & Terrestrial coast	1	0.1
Urban & Cropland & River & Lakes/wetlands	1	0.1
Urban & Cropland & River & Lakes/wetlands & Terrestrial coast	1	0.1
Urban & Forest & Cropland & Marine coast & Terrestrial coast	1	0.1
Urban & Forest & Cropland & River & Lakes/wetlands & Hydraulic	1	0.1
Urban & Forest & Cropland & River & Lakes/wetlands & Terrestrial coast	1	0.1
Urban & Forest & Grassland & Cropland & Hydraulic & Terrestrial coast	1	0.1
Urban & Forest & Grassland & Cropland & Lakes/wetlands & Hydraulic & Terrestrial coast	1	0.1
Urban & Forest & Grassland & Cropland & Lakes/wetlands & Terrestrial coast	1	0.1
Urban & Forest & Grassland & Cropland & River & Lakes/wetlands & Hydraulic & Terrestrial coast & Ocean	1	0.1
Urban & Forest & Grassland & Cropland & River & Marine coast	1	0.1
Urban & Forest & Grassland & Cropland & River & Terrestrial coast	1	0.1
Urban & Forest & Grassland & Lakes/wetlands & Hydraulic	1	0.1
Urban & Forest & Grassland & Lakes/wetlands & Hydraulic & Marine coast & Terrestrial coast	1	0.1
Urban & Forest & Grassland & Lakes/wetlands & Hydraulic & Terrestrial coast	1	0.1
Urban & Forest & Grassland & Marine coast & Terrestrial coast	1	0.1
Urban & Forest & Grassland & River & Lakes/wetlands & Hydraulic & Marine coast & Terrestrial coast	1	0.1
Urban & Forest & Grassland & River & Lakes/wetlands & Hydraulic & Terrestrial coast	1	0.1
Urban & Forest & Grassland & River & Lakes/wetlands & Marine coast	1	0.1
Urban & Forest & Grassland & River & Lakes/wetlands & Marine coast & Terrestrial coast	1	0.1
Urban & Forest & Grassland & River & Lakes/wetlands & Marine coast & Terrestrial coast & Ocean	1	0.1
Urban & Forest & Grassland & Terrestrial coast	1	0.1
Urban & Forest & Hydraulic	1	0.1
Urban & Forest & Lakes/wetlands & Marine coast	1	0.1
Urban & Forest & Lakes/wetlands & Terrestrial coast & Ocean	1	0.1
Urban & Forest & Marine coast & Terrestrial coast & Ocean	1	0.1
Urban & Forest & River & Hydraulic	1	0.1
Urban & Forest & River & Lakes/wetlands & Hydraulic	1	0.1
Urban & Forest & River & Lakes/wetlands & Hydraulic & Marine coast & Terrestrial coast	1	0.1
Urban & Forest & River & Lakes/wetlands & Hydraulic & Terrestrial coast	1	0.1
Urban & Forest & River & Lakes/wetlands & Marine coast & Terrestrial coast	1	0.1
Urban & Forest & River & Marine coast & Terrestrial coast	1	0.1
Urban & Forest & Terrestrial coast & Ocean	1	0.1
Urban & Grassland & Cropland & Hydraulic	1	0.1
Urban & Grassland & Cropland & Hydraulic & Marine coast & Terrestrial coast	1	0.1
Urban & Grassland & Cropland & Lakes/wetlands & Hydraulic & Terrestrial coast	1	0.1
Urban & Grassland & Cropland & River & Hydraulic & Marine coast & Terrestrial coast	1	0.1
Urban & Grassland & Cropland & River & Lakes/wetlands & Hydraulic & Terrestrial coast	1	0.1
Urban & Grassland & Cropland & River & Lakes/wetlands & Marine coast & Terrestrial coast & Ocean	1	0.1
Urban & Grassland & Lakes/wetlands & Marine coast & Terrestrial coast	1	0.1
Urban & Grassland & Lakes/wetlands & Terrestrial coast	1	0.1
Urban & Grassland & River & Lakes/wetlands	1	0.1
Urban & Grassland & River & Lakes/wetlands & Hydraulic	1	0.1
Urban & Grassland & River & Lakes/wetlands & Marine coast & Terrestrial coast	1	0.1
Urban & Grassland & River & Marine coast	1	0.1
Urban & Grassland & River & Terrestrial coast	1	0.1
Urban & Grassland & Terrestrial coast	1	0.1
Urban & Lakes/wetlands & Hydraulic	1	0.1

Environment reported in Q4	Responses	%
Urban & Lakes/wetlands & Hydraulic & Terrestrial coast	1	0.1
Urban & Lakes/wetlands & Marine coast & Terrestrial coast	1	0.1
Urban & Marine coast	1	0.1
Urban & River & Hydraulic & Marine coast & Terrestrial coast	1	0.1
Urban & River & Lakes/wetlands & Hydraulic & Marine coast & Terrestrial coast	1	0.1
Urban & River & Lakes/wetlands & Hydraulic & Terrestrial coast	1	0.1
Urban & River & Lakes/wetlands & Terrestrial coast	1	0.1
Urban & Terrestrial coast	1	0.1

To analyse the data and retain as much information as possible, we created three different datasets for subsequent analysis, each suitable for analysing a specific question. The three datasets were:

- the *original dataset* comprised as many entries as respondents in the survey (1,928 respondents). Each respondent contributed to the database by generating an entry, including those that responded to questions dependent on the environment separately by continental and marine environments. This was used to analyse questions independent of the environment and the taxonomic group, for example question Q7 “Has any negative impact caused by IAS been detected?”.
- the *continental vs. marine* dataset permitted to explore differences between taxonomic groups for continental and marine environments. The responses of those participants that selected both continental and marine environments in Q4 were divided into two different entries, resulting in an equal number of rows as the number of environment categories they reported. This resulted in a higher number of entries than the original dataset (2,091 entries, derived from 1,701 continental + 64 marine + 2*163 continental and marine responses). To prevent pseudo-replication, this dataset was only used for questions dependent on the environment and the taxonomic group (Q5-Q8, Q11-Q15, Q17-Q19), such as question Q5 “How has the number of IAS changed?”.
- the *specific dataset* was created to examine the differences between taxonomic groups for distinct, more specific environment categories than those considered in the previous dataset. In particular, we distinguished between urban areas, terrestrial (including forests, grasslands and croplands), freshwater (comprising rivers, lakes and inner wetlands, and hydraulic structures), coastland (covering terrestrial coastal areas such as cliffs, salt marshes, lagoons, dry beaches, dunes, etc.), and marine environments (including marine coastal areas and oceans). It only considered the responses from participants who selected a single environment (or similar set of environments) in Q4. In comparison to the other datasets, it comprised a considerably reduced number of entries (n=919).

These datasets permitted for a more accurate assessment of data pertaining to diverse environmental categories, encompassing both broad categories (continental or marine) and more specific ones (urban, terrestrial, freshwater, coastland, or marine).
