# Supplementary material for: Management Measures and Trends of Biological Invasions in Europe: A Survey‐Based Assessment of Local Managers
Source: Glob Chang Biol. 2025 Jan 17;31(1):e70028. doi: 10.1111/gcb.70028 (PMC11742469; doi:10.1111/gcb.70028)
Supplement: Supplementary file 2 — Appendix S2. [file GCB-31-e70028-s001.pdf]

This material serves as supplementary content for the research article titled "*Management Measures and Trends of Biological Invasions in Europe: A Survey-Based Assessment of Local Managers*" (DOI: 10.1111/gcb.70028).

Supplementary material 2
Questions of the survey entitled "The management of invasive alien species - practices of local managers"

Q1

Are you involved in the management of invasive alien species (IAS)* anywhere in Europe?

*An invasive alien species (IAS) is a non-native/alien species whose introduction or spread has been found to threaten or adversely affect biodiversity, ecosystem functioning, services, or human well-being.

[ ] Yes

[ ] No

[IF "Yes" is selected in Q1 users go further in the survey and, then, a message appears:]

Note that most of the questions are not mandatory, so you can answer as many of the questions as you wish. Blank answers will be treated as "**Not applicable**". Privacy Individual responses will be anonymized, so that they cannot be linked to the person providing the information. More information about the privacy policy can be found in the footnote.

Q2

Select the country of the area you manage.

Q3

Select the region of the area you manage.

[In some small countries where NTUS2 is very small a message appears:]

For coherence we chose NUTS2, the basic regions for the application of regional policies for all countries. If you are active in IAS management at a higher spatial scale (entire region, country), please choose "not applicable". Later in the survey you will have the opportunity to provide more information about this.

Q4

Select the environments that characterize your management area most accurately.

Select as many options as necessary.

[ ] Urban areas

[ ] Forestry (forests, shrubland, sparsely vegetated land, etc.)

[ ] Grasslands

[ ] Croplands

[ ] Rivers

[ ] Lakes and inner wetlands

[ ] Hydraulic structure (channels, dams, etc.)

[ ] Terrestrial coastal areas (cliffs, salt marshes, lagoons, dry beaches, dunes, etc.)

[ ] Marine coastal areas (intertidal zone, estuaries, shelf, etc.)

[ ] Oceans

Q5

How has the number of IAS changed?*

Respond according to what has happened in the area you manage since 2015 for the different taxonomic groups.

If you started to work in the area later, answer according to all the information you have.

*If you have not collected data, please answer according to your perception.

	Decreased	No change	Increased	Do not know	Not applicable
Plants	[ ]	[ ]	[ ]	[ ]	[ ]
Invertebrates	[ ]	[ ]	[ ]	[ ]	[ ]
Vertebrates	[ ]	[ ]	[ ]	[ ]	[ ]
Other (specify, e.g. “fungi”):	[ ]	[ ]	[ ]	[ ]	[ ]

Q6

How has the area occupied by the IAS changed?*

Respond according to what has happened in the area you manage since 2015 for the different taxonomic groups.

*If you have not collected data, please answer according to your perception.

	Decreased	No change	Increased	Do not know	Not applicable
Plants	[ ]	[ ]	[ ]	[ ]	[ ]
Invertebrates	[ ]	[ ]	[ ]	[ ]	[ ]
Vertebrates	[ ]	[ ]	[ ]	[ ]	[ ]
Other (specify, e.g. “fungi”):	[ ]	[ ]	[ ]	[ ]	[ ]

Q7

Has any negative impact caused by IAS been detected?*

Respond according to what has happened in the area you manage since 2015.

*If you have not collected data, please answer according to your perception.

	Yes	No	Do not know	Not applicable
Impacts on biodiversity	[ ]	[ ]	[ ]	[ ]
Impacts on economic activities	[ ]	[ ]	[ ]	[ ]
Impacts on human health	[ ]	[ ]	[ ]	[ ]
Impacts on ecosystem services	[ ]	[ ]	[ ]	[ ]
Other (specify):	[ ]	[ ]	[ ]	[ ]

[IF "Yes" is selected in "Impacts on biodiversity" in Q7 then Q8 appears:]

Q8

How have the (negative) impacts on the biodiversity caused by IAS changed?*

Respond according to what has happened in the area you manage since 2015 for the different taxonomic groups.

*If you have not collected data, please answer according to your perception.

	Decreased	No change	Increased	Do not know	Not applicable
Plants	[ ]	[ ]	[ ]	[ ]	[ ]
Invertebrates	[ ]	[ ]	[ ]	[ ]	[ ]
Vertebrates	[ ]	[ ]	[ ]	[ ]	[ ]
Other (specify, e.g. "fungi"):	[ ]	[ ]	[ ]	[ ]	[ ]

Q9

Which management measures have been implemented?

Respond according to what has happened in the area you manage since 2015.

Select as many options as necessary.

[ ] Monitoring. Field work to detect and report new IAS, surveillance of existing invaded sites, detection of new invaded sites, surveillance of pathways, etc.

[ ] Prioritisation. Evaluation of IAS risks, establishment of management priorities regarding the species and sites where measures should be taken, etc.

[ ] Prevention. Measures to prevent the introduction and spread of IAS such as public awareness activities, bans of/taxes for IAS possession, etc.

[ ] Eradication and control. Rapid eradication of newly introduced IAS and control of established populations by means of physical, chemical or biological actions, etc.

[ ] Restoration. Measures to improve the environmental quality of sites after IAS removal, e.g., planting of native species or decontamination of water bodies, soils rehabilitation, etc.

Q10

Drag and drop the previous management measures according to the time dedicated to them (in decreasing order with 1 meaning higher dedication).

Respond according to what has happened in the area you manage since 2015.

**Available categories:**	**Ranked categories:**
Monitoring	1. __________________
Prioritisation	2. __________________
Prevention	3. __________________
Eradication and control	4. __________________
Restoration	5. __________________

*[IF "**Monitoring**" in Q10 is selected in Q9 then questions related to "Monitoring of invasive alien species" appear:]*

Q11

Has the number of IAS been monitored?

Respond according to what has happened in the area you manage since 2015 for the different taxonomic groups.

	**Yes**	**No**	**Do not know**	**Not applicable**
Plants	[ ]	[ ]	[ ]	[ ]
Invertebrates	[ ]	[ ]	[ ]	[ ]
Vertebrates	[ ]	[ ]	[ ]	[ ]
Other (specify, e.g. "fungi"):	[ ]	[ ]	[ ]	[ ]

Q12

Has the area occupied by IAS been monitored?

Respond according to what has happened in the area you manage since 2015 for the different taxonomic groups.

	**Yes**	**No**	**Do not know**	**Not applicable**
Plants	[ ]	[ ]	[ ]	[ ]
Invertebrates	[ ]	[ ]	[ ]	[ ]
Vertebrates	[ ]	[ ]	[ ]	[ ]
Other (specify, e.g. "fungi"):	[ ]	[ ]	[ ]	[ ]

Q13

Have the impacts caused by IAS been monitored?

Respond according to what has happened in the area you manage since 2015.

	Yes	No	Do not know	Not applicable
Impacts on biodiversity	[ ]	[ ]	[ ]	[ ]
Impacts on economic activities	[ ]	[ ]	[ ]	[ ]
Impacts on human health	[ ]	[ ]	[ ]	[ ]
Impacts on ecosystem services	[ ]	[ ]	[ ]	[ ]
Other (specify):	[ ]	[ ]	[ ]	[ ]

*[IF "**Prioritisation**" in Q10 is selected in Q9 then questions related to "Prioritisation of invasive alien species" appear:]*

Q14

Has a priority list of IAS been established?

Respond according to what has happened in the area you manage since 2015 for the different taxonomic groups.

	Yes	No	Do not know	Not applicable
Plants	[ ]	[ ]	[ ]	[ ]
Invertebrates	[ ]	[ ]	[ ]	[ ]
Vertebrates	[ ]	[ ]	[ ]	[ ]
Other (specify, e.g. "fungi"):	[ ]	[ ]	[ ]	[ ]

Q15

Have priorities for managing the different invaded sites been established?

Respond according to what has happened in the area you manage since 2015 for the different taxonomic groups.

	Yes	No	Do not know	Not applicable
Plants	[ ]	[ ]	[ ]	[ ]
Invertebrates	[ ]	[ ]	[ ]	[ ]
Vertebrates	[ ]	[ ]	[ ]	[ ]
Other (specify, e.g. "fungi"):	[ ]	[ ]	[ ]	[ ]

*[IF "**Prevention**" in Q10 is selected in Q9 then questions related to "Prevention of invasive alien species" appear:]*

Q16

Which measures have been applied to prevent the introduction or spread of IAS?

Respond according to what has happened in the area you manage since 2015.

Select as many options as necessary.

[ ] Raising public awareness. Public education campaigns to raise awareness about the restrictions and the risks associated to IAS, guides to identify exotic species, information on non-invasive species that can be used in place of IAS, etc.

[ ] Volunteer-based early detection and reporting. Coordinate citizen participation in the field to find, track, describe, photograph or collect samples of IAS and report occurrences, etc.

[ ] Volunteer-based removal efforts. Coordinate citizen participation in the field at local removal efforts or facilitate the removal of IAS from private properties, etc.

[ ] Decontamination of vehicles and/or equipment. Inspections of and/or stations to clean vehicles (e.g. vessels, boats, cars, motorcycles, bicycles, etc.) or equipment (e.g., fishing rod, hiking boats, etc.) when entering or exiting recreational areas.

[ ] Promote non-invasive species. Promote the use of native or non-invasive species in gardening in public or private spaces, zoos, animal parks, etc.

[ ] Guidance on eradication or control of IAS. Provide assistance to citizens interested in handing in an exotic species or removing exotics plants from their properties, provide advice for keeping and handling exotic species, etc.

[ ] Other (specify):

*[IF “**Control and eradication**” in Q10 is selected in Q9 then questions related to “Control and eradication of invasive alien species” appear:]*

Q17

How have the actions aimed to control IAS changed?

Respond according to what has happened in the area you manage since 2015 for the different taxonomic groups.

	There have not been any	Decreased	No change	Increased	Do not know	Not applicable
Plants	[ ]	[ ]	[ ]	[ ]	[ ]	[ ]
Invertebrates	[ ]	[ ]	[ ]	[ ]	[ ]	[ ]
Vertebrates	[ ]	[ ]	[ ]	[ ]	[ ]	[ ]
Other (specify, e.g. “fungi”):	[ ]	[ ]	[ ]	[ ]	[ ]	[ ]

Q18

Has any IAS been eradicated?

Respond according to what has happened in the area you manage since 2015 for the different taxonomic groups.

Select as many options as necessary.

	Yes, complete removal*	Partially, removal of populations**	Tried without success	No	Do not know	Not applicable
Plants	[ ]	[ ]	[ ]	[ ]	[ ]	[ ]

	Yes, complete removal*	Partially, removal of populations**	Tried without success	No	Do not know	Not applicable
Invertebrates	[ ]	[ ]	[ ]	[ ]	[ ]	[ ]
Vertebrates	[ ]	[ ]	[ ]	[ ]	[ ]	[ ]
Other (specify, e.g. “fungi”):	[ ]	[ ]	[ ]	[ ]	[ ]	[ ]

***Yes: complete removal of at least one IAS over the whole area that you manage.**
****Partially: removal of at least one population in the area that you manage.**

Q19

Has any eradication been done before the establishment of populations? (rapid eradication)

Respond according to what has happened in the area you manage since 2015 for the different taxonomic groups.

	Yes	No	Do not know
Plants	[ ]	[ ]	[ ]
Invertebrates	[ ]	[ ]	[ ]
Vertebrates	[ ]	[ ]	[ ]
Other (specify, e.g. “fungi”):	[ ]	[ ]	[ ]

Q20

Select the funding sources you have received for the management of IAS in your area since 2015.

Select more than one if applicable.

[ ] European Union

  [ ] Common agricultural policy (CAP)

  [ ] European Regional Development Fund (ERDF)

  [ ] Cohesion Fund (CF)

  [ ] LIFE projects

  [ ] Horizon Europe (Horizon 2020, Horizon 2030, European Green Deal, etc.)

  [ ] European Maritime and Fisheries Fund (EMFF)

  [ ] Other (specify):

[ ] National public funds

[ ] Regional public funds

[ ] Local public funds

[ ] Private companies

[ ] Private donations

[ ] Non-governmental organization (NGOs) or Non-profit organization (NPOs)

  [ ] International

  [ ] National

  [ ] Regional

[ ] Work contributions (volunteers)

[ ] Other (specify):

Q21

Use this space to share any additional information that you consider relevant about IAS management.

______*free text*_____

Q22

What is the size of the area you manage?

[ ] < 100 km² (10.000 ha)

[ ] 100 - 300 km² (10.000 - 30.000 ha)

[ ] 300 - 500 km² (30.000 - 50.000 ha)

[ ] > 500 km² (50.000 ha)

Q23

Is your management area protected?

[ ] Yes

[ ] No

[ ] Partially

*[IF "**Yes**" is selected in Q26 then Q27 appears:]*

Q24

Specify the protection figure of the area you manage.

______*free text*_____

Q25

Select your affiliation.

Select only one option.

[ ] Public administration

  [ ] Natural protected area

  [ ] Local administration

[ ] Supramunicipal administration

[ ] Regional administration

[ ] National administration

[ ] Non-governmental organization (NGOs) or Non-profit organization (NPOs)

[ ] Private commercial

[ ] Research

[ ] University

[ ] Non-university institution

[ ] Other (specify):

Q26

Select your professional category.

[ ] Executive management (directors)

[ ] Mid-level management (team leaders)

[ ] Professional technician

[ ] Supporting technician

[ ] Other (specify):

Q27

Select the number of years you have been working on IAS management.

[ ] < 1 year

[ ] 1-5 years

[ ] 6-10 years

[ ] 11-20 years

[ ] >20 years
